# Single-Cell Profiling Reveals the Impact of Genetic Alterations on the Differentiation of Inflammation-Induced Murine Colon Tumors

**DOI:** 10.3390/cancers16112040

**Published:** 2024-05-28

**Authors:** Ahmed H. Ghobashi, Rosie Lanzloth, Christopher A. Ladaika, Ashiq Masood, Heather M. O’Hagan

**Affiliations:** 1 Genome, Cell, and Developmental Biology Graduate Program, Department of Biology, Indiana University Bloomington, Bloomington, IN 47405, USA; 2 Medical Sciences Program, Indiana University School of Medicine, Bloomington, IN 47405, USA; 3 Indiana University Melvin and Bren Simon Comprehensive Cancer Center, Indianapolis, IN 46202, USA; 4 Department of Medicine, Indiana University School of Medicine, Indianapolis, IN 46202, USA; 5 Department of Medical and Molecular Genetics, Indiana University School of Medicine, Indianapolis, IN 46202, USA

**Keywords:** genetic mutations, *BRAF* mutation, MSI, colon tumor differentiation, WNT signaling

## Abstract

**Simple Summary:**

Chronic inflammation is one of the major risk factors for colorectal cancer development. This study aims to determine the effect of genetic mutations on inflammation-induced colon tumor cell heterogeneity and differentiation. Using single-cell approaches, we found that the addition of *BRAF*^V600E^ to *Apc* mutant mice (BLM) increased colon epithelial tumor differentiation. Additionally, we found that BLM epithelial tumors had increased expression of revival colon stem cell (RevCSC) markers and reduced WNT signaling compared to *Apc* mutant epithelial tumors (Min). In contrast, the loss of *Msh2* in Min mice (MSH2KO) increased epithelial tumor stem cell populations with increased WNT signaling compared to Min epithelial tumor. We also identified additional potential regulators of BLM epithelial tumor differentiation such as CDX2 and NDRG1.

**Abstract:**

Genetic mutations and chronic inflammation of the colon contribute to the development of colorectal cancer (CRC). Using a murine model of inflammation-induced colon tumorigenesis, we determined how genetic mutations alter colon tumor cell differentiation. Inflammation induced by enterotoxigenic *Bacteroides fragilis* (ETBF) colonization of multiple intestinal neoplasia (Min^ApcΔ716/+^) mice triggers loss of heterozygosity of *Apc* causing colon tumor formation. Here, we report that the addition of *BRAF*^V600E^ mutation (*BRAF*^F-V600E^*Lgr5*^tm1(Cre/ERT2)Cle^Min^ApcΔ716/+^, BLM) or knocking out *Msh2* (*Msh2^LoxP/LoxP^Vil1-cre*Min^ApcΔ716/+^, MSH2KO) in the Min model altered colon tumor differentiation. Using single-cell RNA sequencing, we uncovered the differences between BLM, Min, and MSH2KO tumors at a single-cell resolution. BLM tumors showed an increase in differentiated tumor epithelial cell lineages and a reduction in the tumor stem cell population. Interestingly, the tumor stem cell population of BLM tumors had revival colon stem cell characteristics with low WNT signaling and an increase in RevCSC marker gene expression. In contrast, MSH2KO tumors were characterized by an increased tumor stem cell population that had higher WNT signaling activity compared to Min tumors. Furthermore, overall BLM tumors had higher expression of transcription factors that drive differentiation, such as *Cdx2*, than Min tumors. Using RNA velocity, we identified additional potential regulators of BLM tumor differentiation such as NDRG1. The role of CDX2 and NDRG1 as putative regulators for BLM tumor cell differentiation was verified using organoids derived from BLM tumors. Our results demonstrate the critical connections between genetic mutations and cell differentiation in inflammation-induced colon tumorigenesis. Understanding such roles will deepen our understanding of inflammation-associated colon cancer.

## 1. Introduction

Colorectal cancer (CRC) is the third most common cause of cancer-related mortalities among males and females in the US [1]. Chronic inflammation is one of the major risk factors for CRC [2], as evidenced by patients with inflammatory bowel disease (IBD) having a higher risk of CRC development than individuals without IBD [3]. Alterations in the gut microbiota can also contribute to intestinal inflammation and CRC [4]. Enterotoxigenic *Bacteroides fragilis* (ETBF) is a strain of the common anaerobic gut bacteria, *Bacteroides fragilis*, which secretes a metalloprotease toxin and can lead to severe intestinal inflammation. ETBF is also associated with IBD development and increased risk of CRC incidence [4,5,6,7].

In addition to inflammation, genetic mutations play a key role in CRC initiation [8]. Loss of Adenomatous Polyposis Coli (*APC*), which causes hyperactivation of Wingless/Int (WNT)/β-catenin signaling [9] is the initiator event for CRC development. *APC* gene is mutated in almost 85% of sporadic CRC [10,11]. ETBF colonization of multiple intestinal neoplasia (Min*^Apc^*^Δ716/+^) mice, which are heterozygous for mutant *Apc*, results in loss of heterozygosity (LOH) of the wildtype allele of *Apc* triggering colon tumor formation mainly in the distal part of the colon [12,13,14]. Mutation in or inactivation of mismatch repair (MMR) genes, such as MutS homolog 2 (MSH2), which causes microsatellite instability (MSI), is the most common lesion in hereditary nonpolyposis colorectal cancer (HNPCC or Lynch Syndrome) and occurs in 20% of sporadic CRC [8,15]. 56% of MMR-deficient CRCs also harbor *APC* mutations [16]. We developed a mouse model in which mice have intestine-specific *Msh2* deletion driven by villin-cre and an *Apc* mutation (*Msh2^LoxP/LoxP^Vil1-cre*Min^ApcΔ716/+^, MSH2KO) [12]. We previously showed that MSH2KO tumors had a high percentage of MSI compared to Min tumors [12], which is consistent with the observation that MSI CRCs display a loss of expression of at least one MMR protein [17,18]. *BRAF*-activating mutations, which lead to the activation of the MAPK pathway, occur in almost 10% of CRC [19]. Almost 50% of *BRAF* mutant CRC exhibit hyperactivation of WNT signaling [20]. *BRAF* mutant CRC is characterized by poor overall survival and limited response to chemotherapies [21]. Even though MSI is associated with *BRAF* mutation in CRC, 25–54% of *BRAF* mutant CRC cases are microsatellite stable (MSS), which is characterized by poorer survival compared to *BRAF* mutant-MSI CRC cases [22,23,24,25,26]. To understand how *BRAF* mutation contributes to CRC development, we developed a mouse model in which *BRAF*^V600E^ expression is driven by Lgr5-cre in Min mice (*BRAF*^FV600E^*Lgr5*^tm1(Cre/ERT2)Cle^Min^ApcΔ716/+^, BLM) [27]. We have previously demonstrated that loss of *Msh2* in Min mice increases distal colon tumorigenesis following ETBF colonization compared to Min mice [12]. In contrast, ETBF colonization in BLM mice resulted in an additional new tumor locus in the mid-proximal part of the colon [27], which resembles the right-sided location of *BRAF* mutant tumors observed in CRC patients [21]. Furthermore, BLM tumors exhibited a serrated and mucinous phenotype unlike Min tumors [27]. Our previous works provide evidence for the role of gene mutation–inflammation interactions in inflammation-induced colon cancer tumorigenesis.

The intestinal colon epithelium is a continuously self-renewing tissue organized into defined crypt-villus units. Colon stem cells, located at the base of the crypt, undergo self-renewal and generate transit-amplifying cells, which migrate up the crypt-villus axis and differentiate into absorptive enterocytes and secretory cells [28]. The WNT/β-catenin signaling pathway is essential to maintain stem cell proliferation at the bottom of the crypt [28]. Activating mutations of the WNT/β-catenin pathway, such as loss of *APC*, increases the stem cell population, which is associated with CRC initiation and progression [29]. Genetic mutations and environmental factors, including inflammation, disrupt the balance between stem and differentiated cells and contribute to CRC development.

This study aims to demonstrate the effect of gene mutation–inflammation interactions on colon tumor cell heterogeneity and differentiation. To accomplish this goal, we use single-cell RNA sequencing (scRNA-seq) of colon tumors collected from ETBF colonized Min, MSH2KO, and BLM mice to uncover cell type differences in the tumors at a single-cell resolution. We present here one of the first single-cell comparisons of inflammation-induced tumorigenesis in different genetic backgrounds. Our results demonstrate that genetic mutations induce tumor cell population differences and distinct tumor stem cell differentiation patterns in inflammation-induced colon tumors. Determining how gene mutation–inflammation interactions alter the equilibrium between tumor stem cells and differentiated cells will enhance our understanding of inflammation-associated colon tumorigenesis.

## 2. Materials and Methods

### 2.1. Animal Model

Min^ApcΔ716/+^ mice were handled and inoculated with Enterotoxigenic Bacteroides *fragilis* (ETBF) as in Wu et al. [14]. Msh2^l/l^VC are a result of crossing B6.Cg-MSH2^tm2.1Rak^/J (The Jackson Laboratory; RRID:IMSR_JAX:016231) and B6.Cg-Tg(Vil1-cre) 997Gum/J mice (RRID:IMSR_JAX:004586) to create mice homozygous for MSH2^tm2.1Rak^ and expressing the Vil1-cre transgene. Msh2^l/l^VC/Min (MSH2KO) mice are the result of crossing Msh2^l/l^VC and Min^ApcΔ716/+^ mice for multiple generations. Mice containing LoxP flanked *BRAF*^F-V600E^ (B6.129P2(Cg)-*Braf^tm1Mmcm/J^*; RRID: IMSR_JAX:017837) and leucine-rich repeat-containing G protein-coupled receptor 5 (*Lgr5*) CreERT2 knock-in (*Lgr5*^tm1(Cre/ERT2)Cle^; RRID: IMSR_JAX:008875) were crossed with Min^ApcΔ716/+^ mice to produce *BRAF*^F-V600E^*Lgr5*^tm1(Cre/ERT2)Cle^ Min^ApcΔ716/+^ (BLM) mice. Recombination in mice bearing Lgr5^Cre^ was induced with tamoxifen, as in Barker et al. [30], at 4 weeks of age. All mice were bred and maintained in a specific pathogen-free barrier facility; both males and females were used for all experiments, and mice of different genotypes were cohoused; 6–8 weeks post-ETBF colonization, mice were euthanized, and individual tumors were removed from dissected colons with the aid of a dissecting microscope, pooled, digested, and used for scRNA-seq and organoid derivation as indicated below. Each scRNA-seq dataset is from pooled tumors (Min: 9 tumors, BLM: 6 tumors, BLM2: 5 tumors, MSH2KO: 8 tumors) from one mouse. Alternatively, dissected colons were Swiss rolled, fixed in 10% formalin for 48H, and paraffin-embedded (FFPE). All mouse experiments were covered under an approved Indiana University Bloomington Institutional Animal Care and Use Committee (IACUC) protocol (protocol number 22-010), in accordance with the Association for Assessment and Accreditation of Laboratory Animal Care International.

### 2.2. Tumor Digestion and Single-Cell RNA Sequencing

To obtain single-cell solutions of tumor cells pooled distal (Min, MSH2KO), distal, and mid-proximal (BLM) or mid-proximal (BLM2) tumors were washed in HBSS, incubated in 0.25% trypsin-EDTA for 10 min at 37 °C, followed by the addition of FBS to inactivate the trypsin. Next, the tumors were digested by incubation in Liberase Thermolysin Medium (TM) (0.05 mg/mL; Roche; Basel, Switzerland, no. 05401119001) + DNase (0.2 mg/mL) in DMEM at 37 °C for 2H while rotating. Following washing in HBSS and an additional incubation in 0.25% trypsin-EDTA for 10 min at 37 °C, cells were resuspended in DMEM + 10% FBS and filtered through a 40 μM mesh cell strainer. Cells were then washed with DPBS + 0.1% BSA and viable cells were counted. Cells were resuspended at 1000 cells per ml in DPBS + 0.1% BSA. All single-cell preparations used for sequencing had a viability of >80%. 10,000 cells per sample were targeted for input to the 10X Genomics Chromium system (Pleasanton, CA, USA) using the Chromium Next GEM Single Cell 30 Kit v3.1 (10X Genomics; Pleasanton, CA, USA) at the Indiana University School of Medicine (IUSM) Center for Medical Genomics core. The libraries were sequenced at the IUSM Center for Medical Genomics using a NovaSeq 6000 (Illumina; San Diego, CA, USA) with a NovaSeq S2 reagent kit v1.0 (100 cycles) (Illumina; San Diego, CA, USA) with approximately 450 million read pairs per sample. The remaining cells from each single-cell preparation were used to derive organoids.

### 2.3. Cells and Organoids

HEK293T cells and organoids were maintained in a humidified atmosphere at 37 °C with 5% CO_2_. HEK293T cells were obtained from ATCC and cultured in DMEM 1X (Corning, no. 10-013-CV) with 10% FBS (Corning; Corning, NY, USA, no. 35-015-CV) without antibiotics. Organoids were derived by us for this study from disassociated colon tumors (BLM, BLM2, Min, and MSH2KO) and grown in growth factor-reduced Matrigel plus organoid media (advanced DMEM/F12 (Gibco; Grand Island, NY, USA, no. 12634-010) supplemented with EGF (R&D Systems; Minneapolis, MN, USA, no. 236-EG), Noggin (R&D systems, no. 6057-NG), N2 supplement (Fisher; Waltham, MA, USA, no. 17502048), B27 supplement (Fisher, no. 17504044), HEPES, and Penn/Strep) as in [31].

### 2.4. Generation of Stable Knockdown Organoids

For knockdown of Cdx2 (Sigma-Aldrich; St. Louis, MO, USA, NM_007673, no. TRCN0000055393), Ndrg1 (Sigma, NM_008681, no. TRCN0000238073), and empty vector (EV) TRC2 (Sigma, no. SHC201), the lentiviral shRNA knockdown protocol from The RNAi Consortium Broad Institute was used as in [32]. Briefly, 4 × 10^5^ HEK293T cells were plated on day 1 in DMEM 1X containing 10% FBS. On day 2, cells were transfected with shRNA of interest, EV control, and packaging plasmids. On day 3, the media was replaced with fresh DMEM containing 10% FBS. Approximately 24 h later, media containing lentiviral particles was collected, and fresh DMEM + 10% FBS was added. The added media were collected 24 h later and pooled with media harvested on day 4. The pooled media was then filtered using a 0.45 μm filter and concentrated using a Lenti-X™ Concentrator (Takara; Shiga, Japan, no. 631232). To perform the knockdown, concentrated virus plus polybrene was added to the organoids. Cells were treated with puromycin (1 μg/mL) (Sigma-Aldrich, no. P8833) after 24 h to select for knockdown organoids.

### 2.5. RNA Isolation and Gene Expression

RNA was prepared from organoids using TRIzol followed by cleanup with an RNeasy micro kit (Qiagen; Hilden, Germany, no. 74004) as per the manufacturer’s protocol. The Maxima first strand cDNA synthesis kit (Thermo Fisher; Waltham, MA, USA, no. K1642) for quantitative reverse transcription PCR was used to synthesize cDNA. qPCR was done using TaqMan assays (see Supplementary Table S3 for assays used). The expression of candidate genes was normalized to the expression of a housekeeping gene (*Ppia*).

### 2.6. Immunohistochemistry (IHC)

CDX2 and SAA3 were detected by IHC on 8-mm FFPE colon tissue samples following unmasking in TRIS/EDTA buffer (CDX2) or citrate buffer (SAA3). Anti-CDX2 (ab76541) and anti-SAA3 antibody (ab231680) were applied at a dilution of 1:50, followed by rabbit HRP SignalStain Boost (Cell Signaling Technology; Danvers, MA, USA, no. 8125), rat HRP SignalStain Boost (Cell Signaling Technology, no. 72838), respectively, and DAB substrate (CST, no. 8059). Slides were counterstained in hematoxylin. Tumors stained for CDX2 or SAA3 were scored from 0 to 4. 0: no staining, 1: ≥10% of the tumor epithelial cells were positively stained, 2: 11–33% stained, 3: 34–50% stained, and 4: >50% stained.

### 2.7. Immunofluorescence and Imaging

FFPE colon tissue samples were unmasked in citrate buffer, blocked, and then incubated with anti-SAA3 (ab231680, 1:50), and anti-E-cadherin (Cell Signaling Technology, no. 3195, 1:200) in 1% BSA in PBST overnight at 4 °C. Then, the tissues were incubated with Alexa Fluor (AF)-conjugated secondary antibodies (anti-rat AF488, CST, no. 4416; anti-rabbit AF594, CST, no. 8889) for 2H at room temperature. Images were taken using an EVOS FL Auto microscope (Life Technologies; Carlsbad, CA, USA).

### 2.8. Statistical Analysis

Expression data and IHC are presented as the mean +/− SEM. These data are evaluated by a one-tailed *t*-test and considered statistically significant with a *p* < 0.05.

### 2.9. Computational Analysis

#### 2.9.1. Single-Cell Data Pre-Processing and QC

Read alignment and gene-expression quantification of mouse scRNA-seq data were performed using the Cell Ranger Count pipeline (version 6.1.2, 10X Genomics; Pleasanton, CA, USA). The Cell Ranger pre-built mouse reference package was used for the read alignment (mm10). The filtered feature matrices output was then used to create a Seurat object using the Seurat package v4.3.0.1 [33]. Cells were filtered to include only cells with no more than 20% mitochondrial gene expression and doublets were removed using DoubletFinder v2.0 [34]. The data were normalized, and highly variable genes were identified and scaled using *SCTransform*. Next, dimensionality reduction by principal components (PCs) was calculated using *RunPCA* and to estimate the significant number of PCs to be used *ElbowPlot* function was used. Next, the uniform manifold approximation and projection (UMAP) embedding were calculated and visualized using *RunUMAP* and *DimPlot*. Unsupervised Louvain clustering was carried out using *FindNeighbors* and *FindClusters*. Differentially expressed genes were then defined with *FindAllMarkers* with the Wilcox test.

#### 2.9.2. Data Integration with Batch Correction

In our analysis, we used Seurat (v4.3.0.1) to perform batch-effect correction. 3000 highly variable genes were defined within the 4 mouse samples with the Seurat *FindVariableFeatures* function. We also identified unsupervised integration “anchors” for similar cell states using shared nearest neighbor graphs (*FindIntegrationAnchors*), and then integrated our 4 different datasets using these anchors using *IntegrateData*. The output was then transformed into principal component analysis (PCA) space for further evaluation and visualization.

#### 2.9.3. Subsetting and Visualizing Epithelial Data

To obtain a Seurat object containing only the epithelial cell type of the integrated data, the ‘‘subset’’ function was used. The subset Seurat object goes through Seurat filtering, normalization, and integration workflows as described above. The proportional difference in epithelial cell populations between the two samples was computed using R package’s scProportion (v1.0.0) [35]. Gene set enrichment scores for single cells were computed using escape (v1.12.0) [36]. Diffusion map, diffusion pseudotime, and cell density were computed using *sc.tl.diffmap*, *sc.tl.dpt*, and *sc.pl.embedding_density* respectively, which are implemented through Scanpy (v1.9.6) [37]. The stem cell was annotated manually as a root cell before computing diffusion pseudotime. The average expression levels of different clusters, RevCSC, ProCSC, and proliferation markers were calculated by using the *AddModuleScore* function from the Seurat package.

#### 2.9.4. Gene Ontology Enrichment Analysis

Gene ontology (GO) enrichment was performed using Metascape [38].

#### 2.9.5. RNA Velocity

Spliced/unspliced expression matrices were generated as loom files using Velocyto (v0.17) [39]. Seurat objects were converted into AnnData objects containing the corrected counts, clusters, and UMAP embeddings. Then, the loom files were merged with the AnnData objects and loaded into scVelo (v0.2.1) [40], the ratio of spliced to unspliced reads per cluster was found, and cell velocities were computed. All functions were run with default settings unless otherwise stated. The *scvelo.pp.filter_and_normalize* argument ‘n_top_genes’ was set to 3000, and the ‘n_npcs’ and ‘n_neighbors’ arguments of *scvelo.pp.momentum* were both set to 30. The velocity cell arrows were made with the *scvelo.pl.velocity_embedding* function. The top velocity genes per cluster were discovered using *scvelo.tl.rank_velocity_genes* and plotted using *scvelo.pl.velocity*. *scvelo.tl.velocity_confidence* generated the velocity confidence and length values, and the results were plotted using *scvelo.pl.scatter*. Cell cycle signatures were computed using *scv.tl.score_genes_cell_cycle* and plotted using *scvelo.pl.scatter*. The RNA-velocity analysis was extended by calculating RNA splicing kinetics using a dynamic model using *scv.tl.recover_dynamics* and scv.tl.*velocity* (mode = ‘dynamical’). cluster-specific identification of potential drivers was discovered using *scv.tl.rank_dynamical_genes*. By applying *scv.tl.differential_kinetic_test* to the dynamic model, we were able to discover which cluster exhibited significant RNA splicing kinetics for the *Guca2a* transcript, and the results were plotted using *scvelo.pl.velocity*.

#### 2.9.6. Simulated Gene Perturbation

Simulated gene perturbation was performed using CellOracle [41]. We used gene-regulatory networks (GRN) from mouse scATAC-seq data using *co.data.load_mouse_scATAC_atlas_base_GRN*. Then, GRN data and the BLM2 gene expression matrix were loaded into the CellOracle object using *co.import_TF_data* and *co.import_anndata_as_raw_count*, respectively. We constructed a cluster-specific GRN for each cluster using *oc.get_links* and kept only network edges with *p*-value ≤ 0.01. To simulate gene overexpression or knockout, we perturb the gene expression to 1 or 0, respectively, in the *oc.simulate_shift* function.

## 3. Results

### 3.1. Single-Cell Profiling Identifies Cell Populations in Colon Tumors

**Figure 1:**
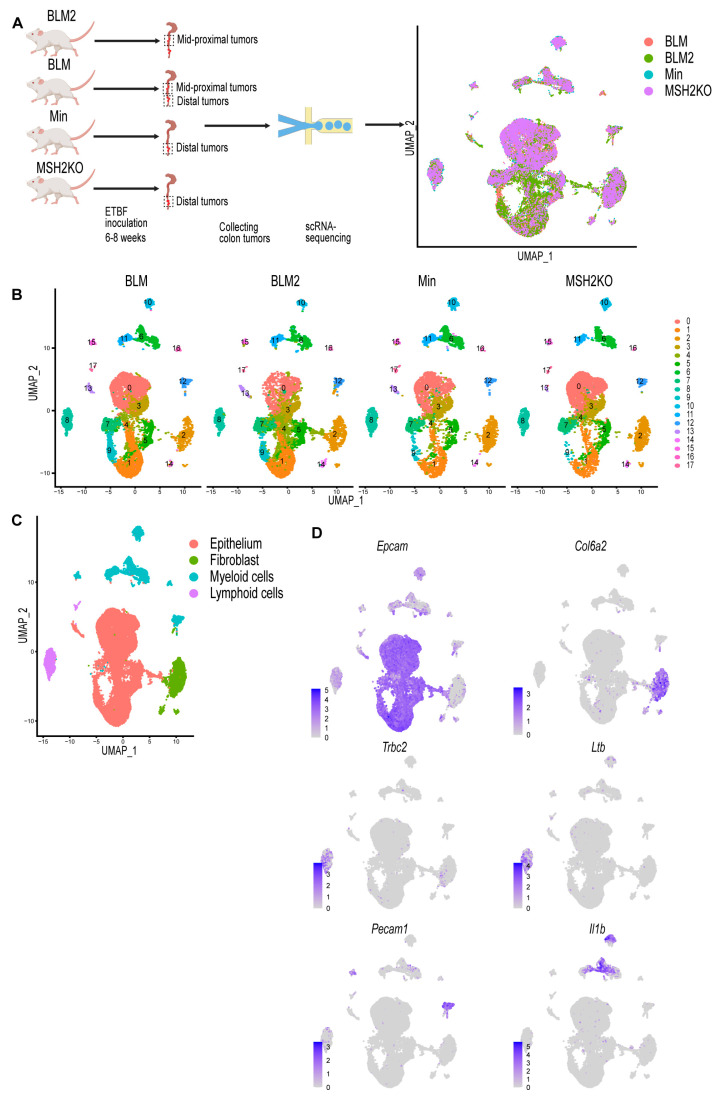
**Single-cell profiling identifies cell populations in colon tumors.** (**A**) Diagrammatic representation of sample preparation and single-cell RNA-sequencing (scRNA-seq) and uniform manifold approximation and projection (UMAP) plot of the four integrated samples. (**B**) UMAP plot of BLM, BLM2, Min, and MSH2KO colon tumor scRNA-seq samples colored by cluster. (**C**) UMAP plot of the four integrated scRNA-seq samples colored by major cell type. (**D**) Feature plot of normalized expression values of marker genes representative of the epithelial, fibroblast, lymphoid, and myeloid cell populations in the combined scRNA-seq samples. Color intensity represents the normalized gene expression.

To investigate whether the *BRAF* mutation or *Msh2* deletion, which is associated with MSI, modifies the cellular composition of ETBF-driven colon tumors, ETBF colonization of BLM, MSH2KO, and Min C57BL/6 mice was performed. For each sample, colon tumors were harvested after 6–8 weeks of ETBF colonization, pooled, and enzymatically digested. Then, scRNA-seq was performed using droplet-based microfluidics (10X Genomics) (Figure 1A). scRNA-seq of tumors from BLM mice from two different experimental cohorts was performed with the samples labeled BLM and BLM2. BLM and BLM2 tumors were harvested after 6 and 8 weeks of ETBF colonization, respectively. Due to the formation of tumors in different regions of the colon in the mouse models used, Min and MSH2KO tumors were harvested from the distal colon, BLM tumors were harvested from the distal and mid-proximal colon, and BLM2 tumors were harvested from the mid-proximal colon only. The number of total single cells initially sequenced from each sample was BLM: 12,273, BLM2: 12,733, MSH2KO: 9618, and Min: 7535.

Quality control was performed by removing cells with mitochondrial content higher than 20% and removing cell doublets. After cell removal, the number of cells for each sample was BLM: 10,062, BLM2: 9946, MSH2KO: 8030, and Min: 6337. Using Seurat [33], data were normalized using *SCTransform*, and 3000 input variable genes were used to identify integration anchors among the four different datasets. Following integration, dimensional reduction and unsupervised Louvain modularity-based clustering were performed resulting in 18 clusters (Figure 1B). Visualizing these subpopulations using uniform manifold approximation and projection (UMAP, Figure 1B) confirmed their distinct identities. To identify different major cell populations (epithelial cells, immune cells, and fibroblasts), we manually assigned class identities based on the expression of well-established marker genes (Figure 1C). Epithelial cells were identified through the expression of *Epcam*, *Krt8*, and *Krt18* (Figure 1D, Supplemental Figure S1). *Col6a2* and *Pdgfrb* were used to identify fibroblasts (Figure 1D, Supplemental Figure S1). *Trbc2*, *Ltb*, and Emb were used to annotate lymphoid cells, and *Pecam1*, *Il1b Cd68*, and *Lyz2* were used to identify myeloid cells Figure 1D, Supplemental Figure S1).

### 3.2. Single-Cell Survey of Colon Tumor Epithelial Cells

**Figure 2:**
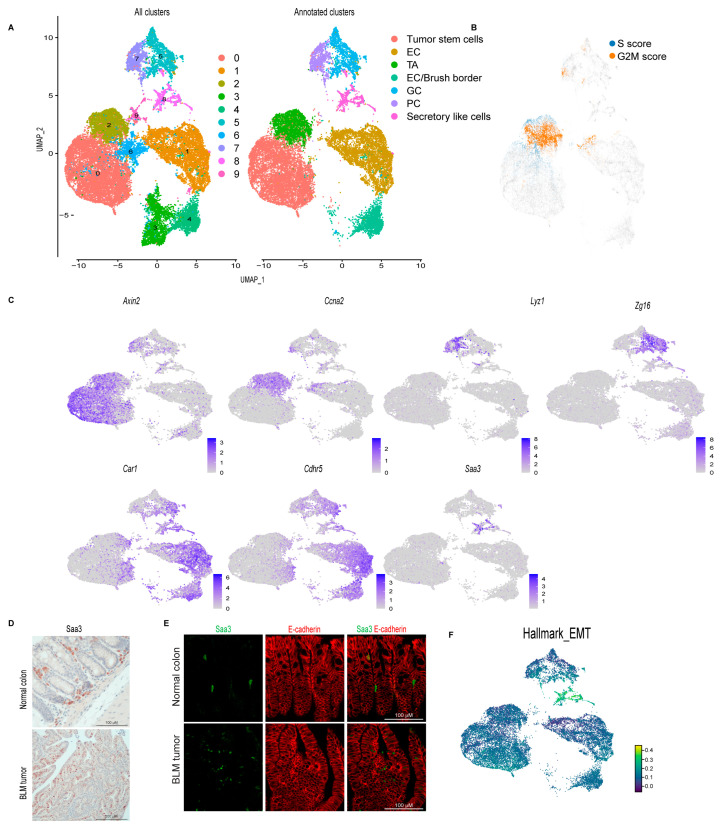
**Single-cell survey of colon tumor epithelial cells.** (**A**) Uniform manifold approximation and projection (UMAP) plot of the combined colon epithelial tumor scRNA-seq data colored by cluster (left, all clusters) and by cell type after filtering out poor quality clusters (right, Annotated clusters; enterocyte cells (EC), transit-amplifying (TA), goblet cells (GC), Paneth cells (PC)). (**B**) UMAP plot of the combined scRNA-seq samples showing the expression of cell cycle signature genes (S score and G2M score). (**C**) Feature plots of normalized expression values of marker genes for the different clusters/cell types. Color intensity represents the normalized gene expression. (**D**) Representative SAA3 IHC of BLM normal colon (Scale bar, 100 μm) and BLM tumor (Scale bar, 200 μm). (**E**) Representative SAA3 (green) and E-cadherin (red) immunofluorescent images of BLM tumor and BLM normal colon (Scale bar, 100 μm). (**F**) UMAP plot of combined BLM, BLM2, Min, and MSH2KO tumor epithelial scRNA-seq data showing the Hallmark EMT score in each cell. Color intensity represents the EMT score.

To focus our study on colon tumor differentiation, we extracted the epithelial cells from the dataset and re-clustered them to produce 10 distinct epithelial cell clusters (Figure 2A). Proliferating cells were identified using cell-cycle signatures (Figure 2B). Seurat FindAllMarkers was utilized to identify transcripts enriched for each cluster, and then each cluster was manually annotated (Supplemental Table S1). We identified a tumor stem cell cluster (cluster 0) with *Axin2*, *Sox4*, *Ccnd2*, and *Clu* expression (Figure 2C, Supplemental Figure S2). Transit-amplifying cells (TA; cluster 2) were enriched for proliferating markers; *Ccna2*, *Pcna*, *Hmgn2*, *Mcm5*, *Top2a*, *Mki67* (Figure 2C, Supplemental Figure S2), which is consistent with the cell-cycle signature result (Figure 2B). Paneth cells (PC; cluster 7) were identified by the expression of the anti-microbial genes *Lyz1*, *Ang4*, *Spink4*, and *Mmp7* (Figure 2C, Supplemental Figure S2). Goblet cells (GC; cluster 5) were marked by the elevated expression of *Zg16*, *Fcgbp*, *Tff3*, *Muc2*, and *Sval1* (Figure 2C, Supplemental Figure S2). Cluster 1 was labeled as enterocyte cells (EC) due to its enrichment with *Car1*, *Fabp2*, *Slc26a3*, and *Ndrg1* (Figure 2C, Supplemental Figure S2). Enterocytes/brush-border cells (cluster 4) were marked by elevated levels of *Cdhr5*, *Car4*, *Aqp8*, *Guca2a*, *Saa1*, and *Espn* (Figure 2C, Supplemental Figure S2). Cluster 8 was characterized by the expression of markers that are normally expressed in fibroblasts such as *Saa3*, *Dcn*, *Lgals1*, *and Bmp4* (Figure 2C, Supplemental Figure S2). Because several of these genes, such as *Saa3* and *Dcn*, have previously been shown to also be expressed in goblet cells [42,43], this cluster was labeled “secretory-like cells” (Figure 2C, Supplemental Figure S2). Additionally, to confirm that this cluster is indeed part of the colon tumor epithelium, we performed IHC for SAA3. While we detected SAA3-positive cells in the epithelial tumor tissue, Saa3-stained cells were mainly stromal cells in the normal colon (Figure 2D). To follow up on our IHC result, we performed immunofluorescence staining for both SAA3 and E-cadherin (an epithelial cell marker). In BLM tumor tissue, unlike normal colon tissue, E-cadherin-positive cells were also positive for SAA3 protein (Figure 2E) further confirming that the *Saa3*-expressing cluster (secretory-like cells) is part of the colon tumor epithelium. Interestingly, SAA3 appeared to be nuclear in the tumor cells but cytoplasmic in the stromal cells in the normal colon (Figure 2D,E). The secretory-like population was also characterized by high expression of genes related to migration and invasion, such as matrix metalloproteinases (MMPs) (Supplemental Table S1) [44]. Consistent with this finding, the epithelial-to-mesenchymal transition (EMT) hallmark was enriched in the secretory-like cell population (Figure 2F). Cluster 9 was removed because the cells were enriched for genes predominantly expressed in immune cells, such as *Lyz2*, *S100a9*, *Il1b*, and *S100a8* (cluster 9, Supplemental Table S1). Clusters 3 and 6 were also excluded because they expressed *AY036118* and *Gm26917*, which are associated with ribosomal RNA contamination (Supplemental Table S1).

### 3.3. BRAF^V600E^ Mutation and Msh2 Deletion Alter Colon Tumor Epithelial Cell Composition

**Figure 3:**
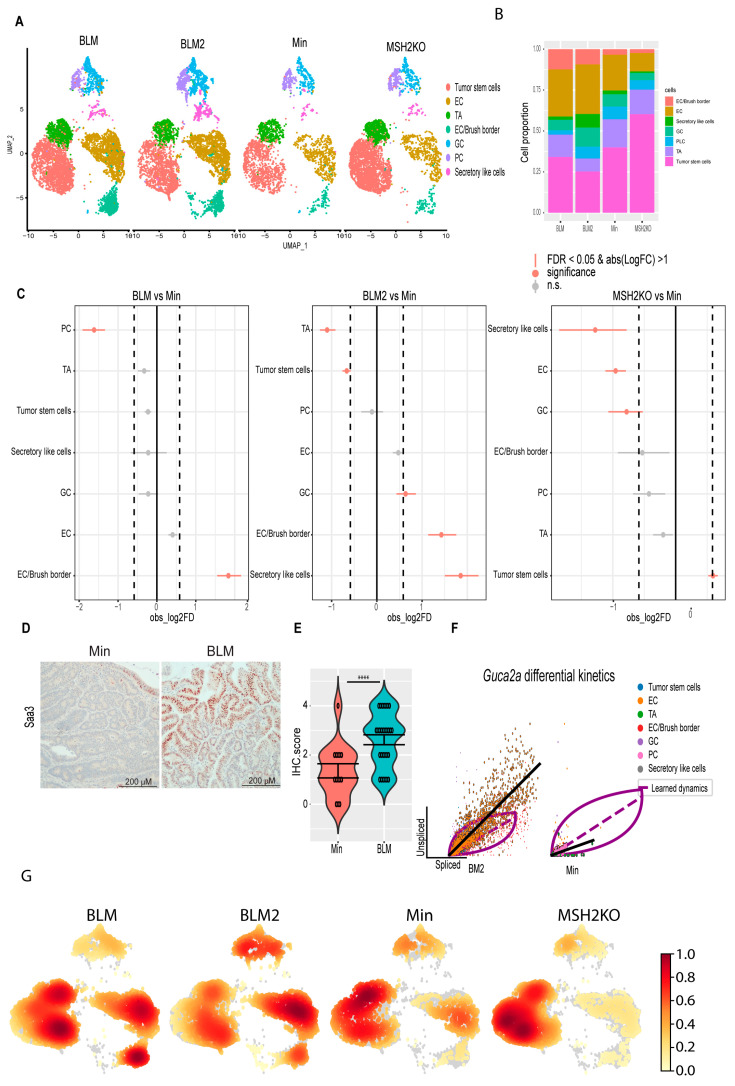
***BRAF* mutation and *Msh2* deletion have different tumor epithelial cellular compositions.** (**A**) Uniform manifold approximation and projection (UMAP) plot of the BLM, BLM2, Min, and MSH2KO colon tumor epithelial cells colored by cell type (enterocyte cells (EC), transit-amplifying (TA), goblet cells (GC), Paneth cells (PC)). (**B**) Stacked barplot for cell proportions in BLM, BLM2, Min, and MSH2KO. (**C**) Relative differences in cell proportions for each cluster between the BLM versus Min, BLM2 versus Min, and MSH2KO versus Min samples. Red dots have an FDR < 0.05 and mean |Log 2-fold change (Log2FC)| > 1 compared with the Min colon tumor (permutation test; n = 10,000). (**D**) Representative SAA3 IHC in BLM and Min colon tumors (Scale bar, 200 μm). (**E**) Quantification of SAA3 IHC stain in (**D**). N = 14 tumors from 4 mice (Min) and 29 tumors from 5 mice (BLM). (**F**) Scatter plot for the unspliced and spliced transcript for *Guca2a* calculated by RNA velocity. The black line represents the significant differential RNA splicing kinetics for enterocytes (BLM2) and Paneth cells (Min) and the purple line represents the overall RNA dynamic. Cells are colored by cell type. (**G**) Embedding density plot of BLM, BLM2, Min, and MSH2KO samples. Colors represent the scaled density values. Significance was determined by paired *t*-test. **** *p* ≤ 0.0001.

To investigate the effect of the expression of the *BRAF^V600E^* mutation or *Msh2* deletion on epithelial cell composition in inflammation-induced colon tumors, we compared the proportion of cells in the different colon tumor cell populations between the different tumor types using Min tumors as a baseline (Figure 3A–C). Interestingly, *BRAF* mutant tumors (BLM and BLM2) were enriched for differentiated cell populations such as secretory-like cells, goblet cells, and EC/brush border cells compared to Min tumors (Figure 3C, BLM vs. Min and BLM2 vs. Min). In contrast, *BRAF* mutant tumors had significantly reduced tumor stem cell and TA cell populations compared to Min tumors (Figure 3C, BLM2 vs. Min). These results agree with other findings suggesting that *BRAF* mutation induces colon epithelial cell differentiation [45]. While *Msh2* deleted tumors had a significantly increased tumor stem cell population, they had reduced differentiated cell populations such as secretory-like cells, enterocytes, and goblet cells in comparison to Min tumors (Figure 3C, MSH2KO vs. Min). Next, we compared the expression of different transcripts that are known to be specific to each lineage. In agreement with more differentiated cells in BLM tumors, BLM tumors had higher co-expression of *Muc2* and *Zg16* (Goblet cells), *Fabp2* and *Slc26a3* (enterocytes), and *Saa3* and *Dcn* (secretory-like cells) than Min and MSH2KO tumors (Supplemental Figure S3A). Consistent with the significant increase of secretory-like cells in BLM2 (Figure 3C, BLM2 vs. Min), tumor cells in BLM tumors showed a significant increase in SAA3 protein levels compared to Min tumors by IHC (Figure 3D,E). Paneth cell markers, *Lyz1* and *Ang4*, were co-expressed more in Min and MSH2KO tumor Paneth cells than BLM tumors (Supplemental Figure S3A), which is consistent with the smaller Paneth cell population in BLM tumors (Figure 3C, BLM vs. Min). Because Paneth cells are involved in antimicrobial activity, we were interested in the expression pattern of other antimicrobial peptides such as REG3G and GUCA2A. Interestingly, while *Reg3g* and *Guca2a* were primarily restricted to Paneth cells in Min and MSH2KO tumors, they were also expressed in enterocytes of BLM tumors (Supplemental Figure S3B). Because *Guca2a* was expressed in multiple lineages, we computed RNA velocity for *Guca2a* to calculate its differential RNA splicing kinetics allowing us to determine which lineages display RNA splicing kinetics for the *Guca2a* transcript in the different tumor types (see methods). We found that in BLM tumors, *Guca2a* exhibited high velocity (high unspliced to spliced RNA ratio) in goblet and enterocyte cells with significant differential RNA splicing kinetics in enterocytes (Fit pval kinetics = 2.29 × 10^−7^) (Figure 3F, BLM2). However, in Min tumors, Paneth cells and TA showed significant differential kinetics for the *Guca2a* transcript (Fit pval kinetics = 5.99 × 10^−6^) (Figure 3F, Min).

To confirm that BLM tumors were more differentiated than Min tumors, we calculated the density of cells in 2D space. Consistent with our findings, BLM2 tumors showed more cell density toward the differentiated enterocyte lineages than Min tumors, while the TA cluster had more cell density in Min tumors (Figure 3G). BLM tumors showed an intermediate phenotype between BLM2 and Min with high cell density in enterocyte and TA clusters, as well as part of the tumor, stem cell population (Figure 3G, BLM). There was also more cell density in the tumor stem cell population in MSH2KO tumors compared to Min and BLM tumors (Figure 3G, MSH2KO). Our findings suggest that expression of mutant *BRAF^V600E^* or deletion of *Msh2* alters cell composition in inflammation-induced colon tumors with increased and decreased differentiated cells in BLM and MSH2KO tumors, respectively.

### 3.4. BRAF^V600E^ Colon Tumors Are More Differentiated Than Min and Msh2 Deleted Tumors

**Figure 4:**
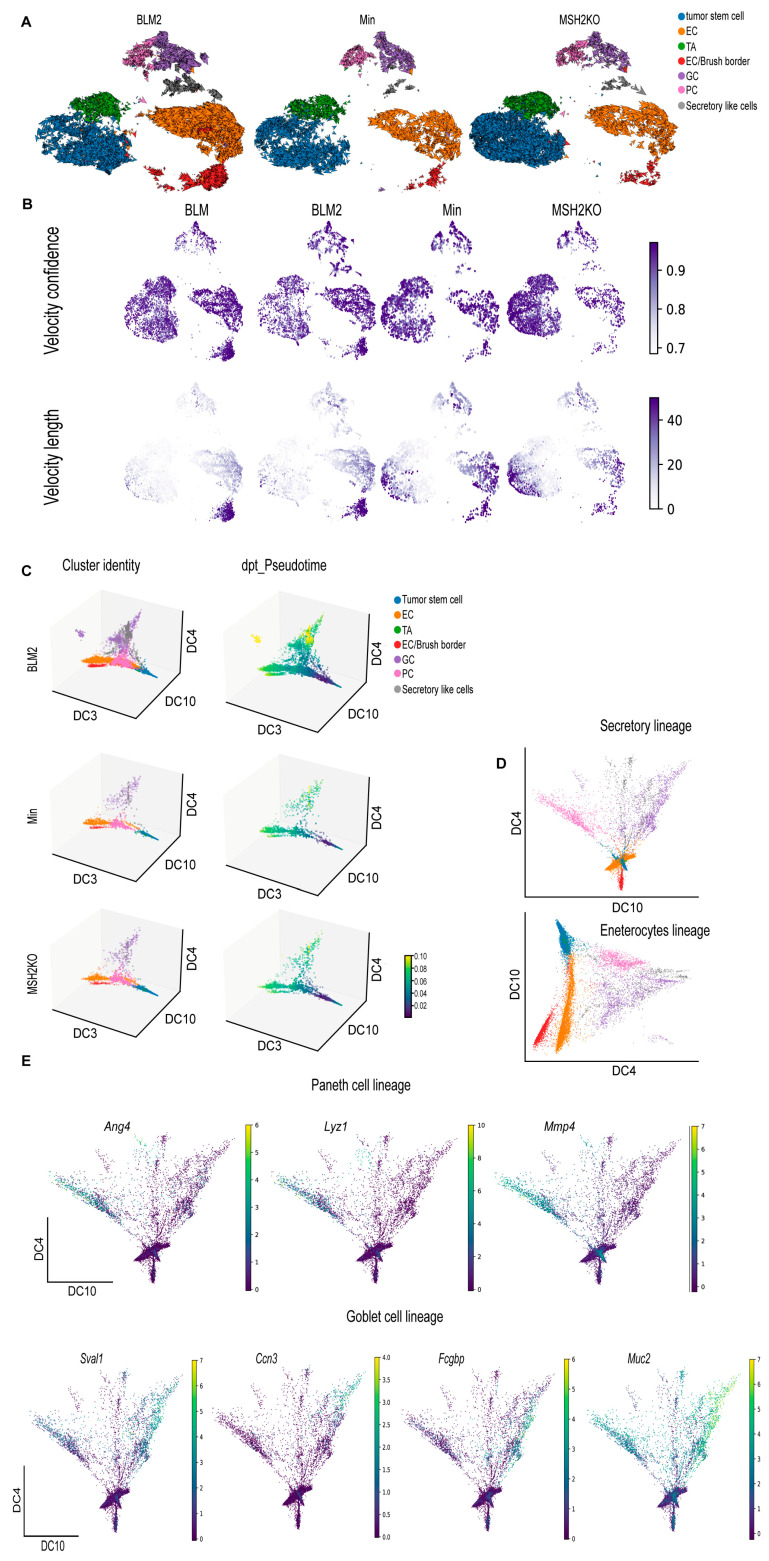
***BRAF* mutant colon tumors are more differentiated than Min and *Msh2* deleted tumors.** (**A**) RNA velocity arrows for individual cells of the BLM2, Min, and MSH2KO tumor epithelial scRNA-seq data colored by cell type, showing inferred differentiation trajectories (enterocyte cells (EC), transit-amplifying (TA), goblet cells (GC), Paneth cells (PC)). (**B**) Uniform manifold approximation and projection (UMAP) plot showing RNA velocity confidence and length in BLM, BLM2, Min, and MSH2KO samples. Color intensity represents the velocity confidence and length values. (**C**) The diffusion-map embeddings of BLM2, Min, and MSH2KO colon tumor epithelial cells are colored by cell type (left, Cluster identity) and diffusion pseudotime (right, dpt_Pseudotime). Diffusion components (DCs) 3, 4, and 10 correspond approximately to the colon tumor differentiation state. Colors represent pseudo-time values. (**D**) Diffusion-map embedding of combined BLM, BLM2, Min, and MSH2KO colon tumor epithelium. DCs 4 and 10 capture secretory lineage differentiation (top) and DCs 3 and 4 capture enterocyte lineage differentiation (bottom). (**E**) Expression of regional markers of Paneth cell lineage (top) and goblet cell lineage (bottom) in the combined BLM, BLM2, Min, and MSH2KO tumor epithelial scRNA-seq data. Colors represent the normalized gene expression.

We hypothesized that the difference in tumor cellular composition among the different datasets is due to differences in colon tumor cell differentiation. To better understand colon tumor epithelial cell differentiation in the different tumor types, computational trajectory analysis was performed. For all samples, a cyclical pattern emerged representing cycling tumor stem cells and TA cells that also branched to secretory lineages and enterocytes (Figure 4A). While the BLM tumors had more cells moving toward differentiated lineages (Figure 4A, BLM2), MSH2KO tumors appeared to have more cycling tumor stem cells in comparison to Min tumors (Figure 4A, MSH2KO). Additionally, we computed velocity confidence and velocity length to predict cell directionality and differentiation speed, respectively [40]. Compared to Min tumors, BLM tumors showed more directionality toward differentiated lineages such as goblet cells, enterocytes, and brush border cells with an increased rate of differentiation at enterocytes and brush border cells (Figure 4B, BLM, and BLM2). MSH2KO tumors showed more directionality and speed toward the tumor stem cell population compared to Min tumors (Figure 4B, MSH2KO).

We next used diffusion maps to place the colon tumor epithelial populations in pseudo-temporal order [46] (Figure 4C, Supplemental Figure S4A). We observed a trajectory from tumor stem cells to the different differentiated lineages (Figure 4C, cluster identity) and captured distinct paths towards enterocytes, goblet cells, secretory-like cells, and Paneth cells (Figure 4C, cluster identity). Importantly, the secretory-like cells were on the same trajectory as the other secretory cell types (goblet and Paneth cells), further supporting our identification of them as a secretory-like population. We also computed Diffusion Pseudotime (dpt) to calculate cell progression during differentiation. Consistent with more epithelial cell differentiation in BLM tumors, we observed more differentiated cells (yellow) on the pseudotime scale in BLM tumors compared to Min tumors (Figure 4C, dpt_pseudotime). Using diffusion components 4 and 10 only, we were able to obtain better separation of the different secretory lineages (Figure 4D, secretory lineages). Improved trajectory separation for enterocytes/brush border cells was accomplished using diffusion components 3 and 4 (Figure 4D, enterocyte lineage). By identifying transcripts that were expressed in different regions of the diffusion map, we were able to associate the expression of genes with cell fate commitment to Paneth cells (*Ang4*, Lyz1, *Mmp7*; Figure 4E), goblet cells (*Sval1*, *Ccn3*, *Fcgbp*, *Muc2*; Figure 4E), secretory-like cells (*Saa3*, *Dcn*, *Bmp4*; Supplemental Figure S4B), and enterocytes and brush border cells (*Saa1*, *Aqp8*, *Cdhr5*, *Muc3*, *Mall*; Supplemental Figure S4B).

### 3.5. BLM and MSH2KO Tumors Have Different Tumor Stem Cell Characteristics

**Figure 5:**
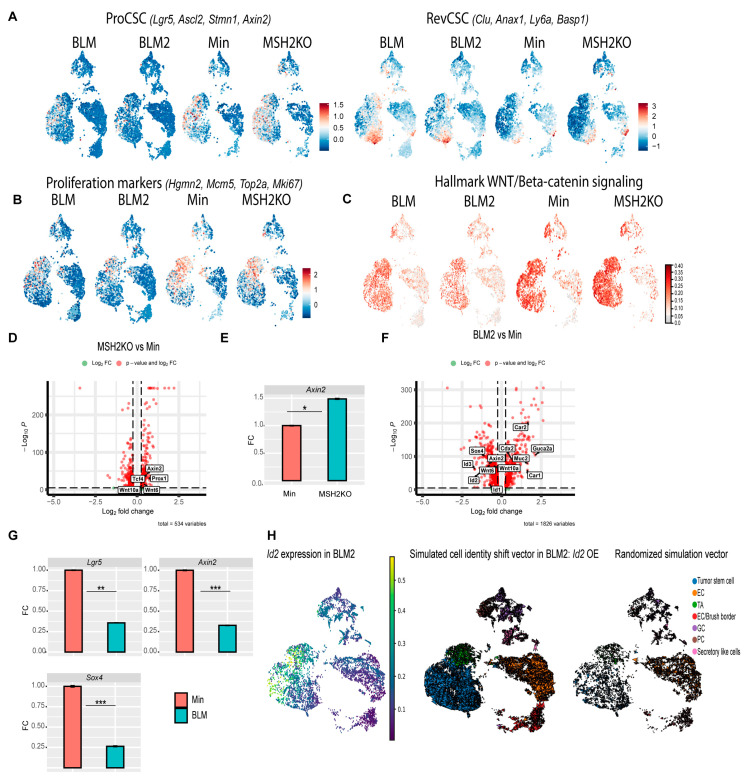
**Tumor stem cells of BLM and MSH2KO colon tumors have low and high WNT signaling activity, respectively.** (**A**) Uniform manifold approximation and projection (UMAP) plot of BLM, BLM2, Min, and MSH2KO tumor epithelial scRNA-seq data showing the expression of RevCSC and ProCSC markers. Color gradient represents the average gene expression. (**B**) UMAP plot of BLM, BLM2, Min, and MSH2KO tumor epithelial scRNA-seq data showing the expression of proliferation markers. Color gradient represents the average gene expression. (**C**) UMAP plot of BLM, BLM2, Min, and MSH2KO tumor epithelial scRNA-seq data showing the Hallmark WNT/β-catenin signaling score in each cell. Color intensity represents the WNT signaling score. (**D**) Volcanoplot of differentially expressed genes (DEGs) in the tumor stem cell populations of MSH2KO versus Min. Dashed lines indicate |log 2FC| > 0.25 and *p* < 0.05. (**E**) Gene expression of the indicated genes by RT-qPCR. Expression of all the genes was normalized to the housekeeping gene *Ppia* and then to Min organoids. Results are represented as the mean of 3 biological replicates +/− SEM. (**F**) Volcanoplot of DEGs in the tumor stem cell populations of BLM2 versus Min. Dashed lines indicate |log 2FC| > 0.25 and *p* < 0.05. (**G**) Gene expression of the indicated genes by RT-qPCR as in C. (**H**) UMAP plot showing the expression of *Id2* in BLM2 tumor epithelial cells (left), RNA velocity arrows for individual cells in simulated *Id2* overexpression in BLM2 colon tumor epithelial cells (middle), and RNA velocity arrows for randomized simulation vector (right). Colors represent the normalized gene expression. Significance was determined by paired *t*-test. * *p* ≤ 0.05, ** *p* ≤ 0.01, *** *p* ≤ 0.001.

Several studies demonstrated that slow-cycling colon stem cells called revival colon stem cells (RevCSCs) are responsible for replenishing the colon after colon damage [47,48,49]. Unlike fast-cycling proliferative colon stem cells (ProCSCs), RevCSCs have high differentiation potential allowing them to repopulate the colon in response to colon damage [48]. MSH2KO and BLM tumors showed high cell density at tumor stem cell and enterocyte clusters, respectively (Figure 3G). Additionally, our data showed that the rate of differentiation in MSH2KO and BLM tumors was highest in tumor stem cell and enterocyte populations, respectively (Figure 4B). Therefore, we hypothesized that colon tumor stem cells would differ between tumor types even though all tumors are from mice on the Min background. Interestingly, the tumor stem cell population of Min and MSH2KO epithelial tumors had a larger number of cells co-expressing markers of ProCSCs (*Lgr5*, *Ascl2*, *Stmn1*, *Axin2*), compared to BLM epithelial tumors (Figure 5A). In contrast, more tumor stem cells of BLM epithelial tumors had co-expression of markers of RevCSCs (*Clu*, *Anxa1*, *Ly6a*, *Basp1*), compared to the tumor stem cell populations of Min and MSH2KO tumors (Figure 5A). Additionally, tumor stem cell and TA populations of Min and MSH2KO epithelial tumors had a larger number of cells co-expressing cell proliferation makers (*Hgmn2*, *Mcm5*, *Top2a*, *Mki67*) compared to BLM epithelial tumors (Figure 5B) consistent with the proliferative nature of ProCSCs [48]. While tumor stem cells of MSH2KO tumors showed high levels of WNT signaling pathway activity compared to Min tumors, tumor stem cells in BLM and BLM2 tumors showed low WNT signaling activity (Figure 5C). These results are consistent with ProCSCs being characterized by high WNT signaling compared to RevCSCs [48,49].

To further explore differences in the tumor stem cells, we calculated differential gene expression between the tumor stem cell clusters in MSH2KO and Min tumors. MSH2KO tumor stem cells had increased expression of WNT and stemness-related genes such as *Axin2*, *Tcf4*, *Wnt6*, *Wnt10a*, and *Prox1* relative to Min tumor stem cells (Figure 5D). *Wnt6* and *Wnt10a* were highly expressed in tumor stem cell populations of MSH2KO tumors compared to Min tumors and were almost absent in BLM tumor stem cells (Supplemental Figure S5A). Additionally, organoids derived from MSH2KO tumors had significantly increased *Axin2* expression compared to organoids derived from Min tumors (Figure 5E). Gene ontology (GO) analysis demonstrated that upregulated genes in MSH2KO tumor stem cells were enriched for the WNT signaling pathway (Supplemental Figure S5B). Consistent with low WNT signaling in RevCSCs, BLM tumor stem cells had decreased expression of WNT/stemness-related genes compared to Min tumors such as *Axin2*, *Id2*, *Sox4*, *Wnt6*, *Wnt10a*, *Id3*, *Prox1*, and *Id1* (Figure 5F, Supplemental Figure S5C). In contrast, in addition to *Clu* and *Ly6a*, the tumor stem cell population of BLM tumors had significant upregulation of differentiation-related genes such as *Guca2a*, *Car1*, *Cdx2*, *Car2*, and *Muc2* (Figure 5F, Supplemental Figure S5C). Additionally, organoids derived from BLM2 tumors had a significantly lower expression of *Lgr5*, *Axin2*, and *Sox4* compared to Min organoids (Figure 5G). Furthermore, upregulated genes in BLM tumor stem cells showed significant enrichment for epithelial cell differentiation and regulation of microvillus length by GO analysis (Supplemental Figure S5D). To computationally determine if the reduction of cycling tumor stem cells in BLM2 is due to the reduced expression of stemness-related genes, we simulated the over-expression (OE) of *Id2*, a gene that was mainly expressed in the tumor stem cells of BLM2 tumors (Figure 5H) and was significantly downregulated in tumor stem cells of BLM tumors compared to Min tumors (Figure 5F, Supplemental Figure S5C). Interestingly, simulated OE of *Id2* in BLM2 caused a shift in TA and differentiated cells toward the tumor stem cell population with more cycling TA and tumor stem cells as compared to a randomized simulation control, which did not alter the trajectory of any cell type (Figure 5H compared to BLM2 in Figure 4A). These findings suggest that increased WNT signaling activity in the tumor stem cell population of MSH2KO tumors contributes to the increased number of tumor stem cells and reduced differentiation in these tumors and that the enrichment of RevCSCs in BLM tumors contributes to their enhanced differentiation.

### 3.6. CDX2 Is Involved in BRAF^V600E^ Colon Tumor epithelial Cell Differentiation

**Figure 6:**
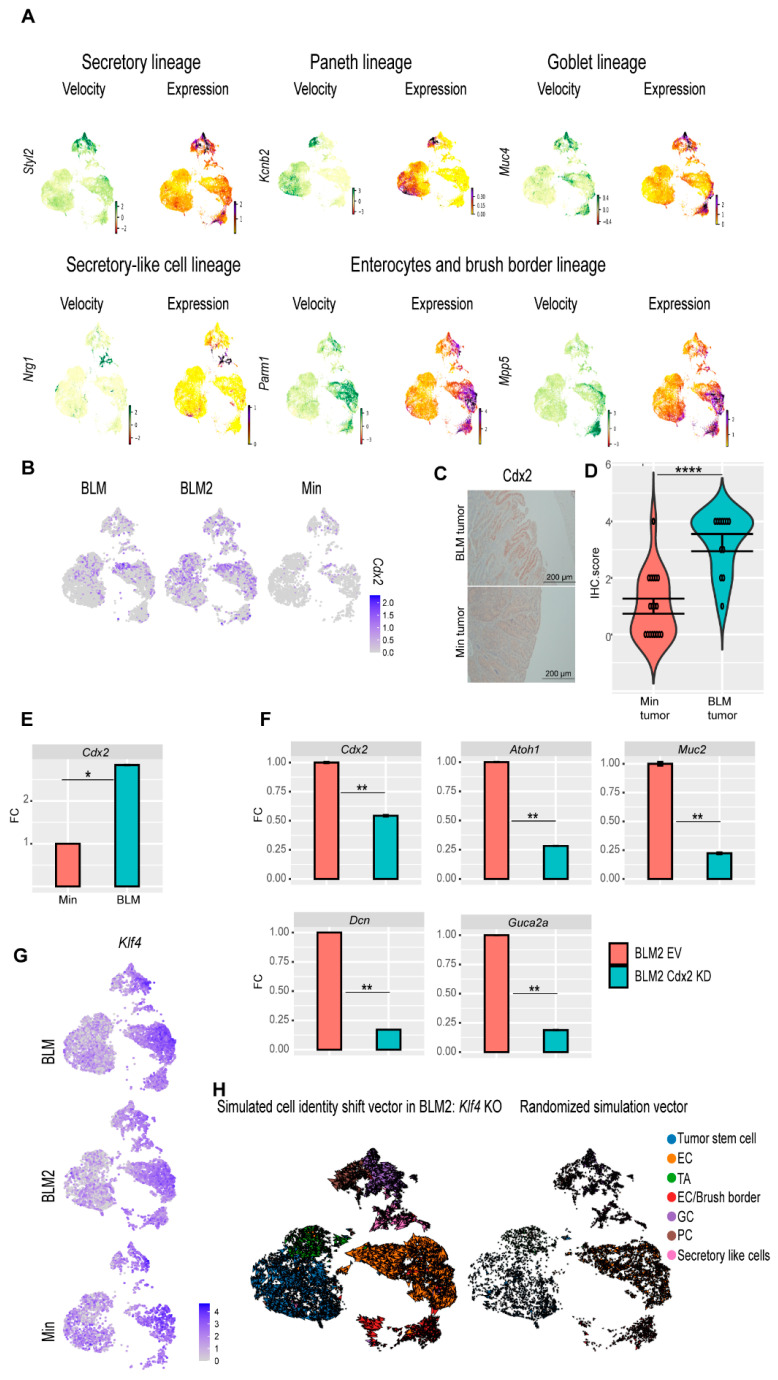
**scRNA-seq reveals genes involved in BLM colon tumor epithelial cell differentiation.** (**A**) Velocity and expression Uniform manifold approximation and projection (UMAP) plots of indicated genes that have differential velocity in the indicated cell lineages in combined tumor epithelial scRNA-seq data. Colors represent normalized gene and velocity expression. (**B**) Feature plot of normalized *Cdx2* expression in BLM, BLM2, and Min colon tumor epithelial cells. Color intensity represents normalized *Cdx2* gene expression. (**C**) Representative CDX2 IHC in BLM and Min colon tumors (Scale bar, 200 μm). (**D**) Quantification of Cdx2 IHC in (**C**). N = 18 tumors from 4 mice (Min) and 12 tumors from 5 mice (BLM). (**E**,**F**) Gene expression of the indicated genes in tumor organoids by RT-qPCR. Expression of all the genes was normalized to the housekeeping gene *Ppia* and then to Min organoids in (**E**) or to empty vector (EV) BLM2 organoids in (**F**). Results are represented as the mean of 3 biological replicates +/− SEM. (**G**) Feature plot of normalized *Klf4* expression in BLM, BLM2, and Min colon tumor epithelium samples. (**H**) UMAP plot showing the RNA velocity arrows for individual cells in simulated *KLF4* knockout in BLM2 colon tumor epithelial cells (left) and RNA velocity arrows for randomized simulation vector (right). Significance was determined by paired *t*-test. * *p* ≤ 0.05, ** *p* ≤ 0.01, **** *p* ≤ 0.0001.

So far, our data suggest that BLM tumors are more differentiated than Min tumors. To identify additional genes whose velocity drives toward different differentiation trajectories, cluster-specific differential velocity expressions were computed. For the secretory lineage, individual genes such as *Spdef* were identified, which are known to regulate secretory cell (Paneth and goblet cell) specification in the normal intestine [50], as well as additional novel regulators such as *Sytl2* and *Pld1* (Figure 6A, Secretory lineage, Supplemental Table S2). Genes were identified that are associated with Paneth cell differentiation trajectories such as *Kcnb2*, *Hepacam2*, *Foxa3*, *Ern2*, and *Klf7* suggesting these genes may be novel regulators of Paneth cell differentiation (Figure 6A, Paneth lineage, Supplemental Table S2). *Muc2* and *Stim1*, which are known to regulate goblet cell differentiation [51,52], as well as other novel goblet cell regulators such as *Muc4*, *Dstn*, and *Muc13*, were identified (Figure 6A, Goblet lineage, Supplemental Table S2). Secretory-like cell regulators were identified such as *Nrg1*, *Piezo2*, and *Ncam1* (Figure 6A, Secretory-like cell lineage, Supplemental Table S2). *Parm1*, *Mpp5*, *Muc3*, *Syk*, *Ascl3*, *Prag1*, *Cdh17*, and *Sgk2* were identified as potential regulators for enterocyte cell and enterocyte/brush border cell specification (Figure 6A, Enterocytes and brush border lineage, Supplementary Table S2).

The CDX2 transcription factor plays an essential role in the development of the intestinal epithelium [53,54]. Because tumor stem cells of BLM tumors showed increased expression of *Cdx2* (Figure 5D), we focused on CDX2 as a potential regulator of BLM tumor differentiation. BLM tumors had more *Cdx2*-expressing cells than Min tumors (Figure 6B). Furthermore, by IHC, BLM tumor tissue had higher levels of CDX2 protein than Min tumors (Figure 6C,D), whereas normal Min and BLM colon tissue had similar levels of CDX2 protein (Supplemental Figure S6A). These findings suggest that inflammation-induced colon tumorigenesis induces loss of *Cdx2* expression in Min mice. Additionally, BLM organoids had significantly higher expression of *Cdx2* than Min organoids (Figure 6E). To test the effect of CDX2 loss on differentiation, we knocked down *Cdx2* in organoids derived from BLM and BLM2 tumors (Figure 6E, Supplemental Figure S6B). Interestingly, knocking down *Cdx2* significantly reduced the expression of *Atoh1* (Secretory cells), *Muc2* (Goblet cells), *Dcn* (secretory-like cells), and *Guca2a* (Goblet and enterocyte cells) (Figure 6F, Supplemental Figure S6B). These results suggest that the expression of *Cdx2* in BLM tumor cells contributes to their differentiation.

In addition to CDX2, KLF4 is another transcription factor that plays an essential role in colon epithelium differentiation [55]. *Klf4* had the highest levels of expression in differentiated cells, including goblet, secretory-like cells, and enterocyte cells (Figure 6G). To test if disruption of *Klf4* expression affects BLM tumor differentiation, we simulated *Klf4* knockout (KO). Simulated *Klf4* KO changed the directionality of differentiated cells of BLM2 tumors, shifting their direction toward the tumor stem cells (Figure 6H, compared to BLM2 in Figure 4A). This finding suggests that *Klf4* expression in BLM tumors may also contribute to BLM epithelial tumor differentiation.

To identify other potential driver genes for BLM tumor differentiation, we computed transcriptional dynamics using RNA velocity, which identified *Ndrg1* as a potential regulator of BLM tumor epithelial cell differentiation. NDRG1 has previously been shown to be involved in the differentiation of adipocytes and macrophages [56,57]. The enterocyte clusters had the highest level of *Ndrg1* expression (Figure S6C, Expression). *Ndrg1* also had high velocity in enterocytes, and almost all the enterocyte populations showed high *Ndrg1* velocity in BLM and Min tumors (Figure S6C, Velocity). Interestingly, *Ndrg1* had high velocity in the tumor stem cells of BLM tumors, while in Min tumors, *Ndrg1* only started to show velocity in differentiated enterocytes and goblet cells (Figure S6C, Velocity). This observation suggests that the high velocity of *Ndrg1* in tumor stem cells might cause stem cells to differentiate in BLM tumors. Additionally, knocking down *Ndrg1* in BLM tumor organoids reduced secretory progenitor marker gene expression (Figure S6D; *Sox4*, *Atoh1*, *Dll1*, *Notch1*), but increased enterocyte marker gene expression (Figure S6D; *Cdhr2*, *Aqp8*). These results suggest that Ndrg1 may be an additional regulator of BLM tumor epithelial cell differentiation.

## 4. Discussion

Recent advances in scRNA-seq technology have allowed for the evaluation of the intestinal epithelium, providing insights into the complexity and diversity of intestinal epithelium cell populations [58,59]. However, limited studies have examined the role of gene mutation and inflammation on cellular heterogeneity of intestinal epithelial tumors at a single-cell resolution. We previously demonstrated the impact of genetic mutations on phenotypic and molecular characteristics of inflammation-induced colon tumorigenesis [12,27]. Many studies have demonstrated that genetic mutations and tumor heterogeneity play an enormous role in the effectiveness of chemotherapeutic treatments [60,61]. Therefore, utilizing scRNA-seq, we focused on evaluating the changes in the cellular composition and differentiation of inflammation-induced murine colon tumors from three different genetic backgrounds (Min, BLM, and MSH2KO). We used ETBF colonization to induce colon inflammation because ETBF colonization of colon mucosa is associated with CRC incidence in humans [4,5,6,7]. Importantly, ETBF induces LOH of *Apc* to trigger colon tumor formation without causing additional genetic mutations making it ideal to study inflammation-genetic interactions [13]. Through our single-cell analysis of the different tumor types, we determined that the expression of *BRAF^V600E^* or *Msh2* deletion in Min mice altered the differentiation of inflammation-induced colon tumors. We determined that the expression of mutant *BRAF* increases the differentiation of colon tumor cells and that loss of *Msh2* reduces colon tumor cell differentiation, increasing the tumor stem cell population compared to Min tumors.

The expression of mutant BRAF has been reported to increase colon epithelium differentiation [45,62]. For example, expression of *BRAF*^V600E^ has been shown to trigger intestinal stem cell differentiation [45,62]. Previously, we demonstrated that ETBF-induced *BRAF* mutant colon tumors are characterized by a mucinous phenotype [27]. Here, we demonstrated that BLM tumors are more differentiated than Min tumors with an increase in enterocytes, goblet cells, and secretory-like cell populations. The increase in both goblet and secretory-like cells may contribute to the mucinous phenotype in BLM tumors. The expression of *BRAF*^V600E^ in intestinal epithelium reduces the level of intestinal stem cell markers *OLFM4* [45]. We demonstrated here that the tumor stem cells of BLM tumors exhibited low WNT pathway activity compared to Min tumors. Interestingly, BLM tumor stem cells had higher expression of RevCSC markers such as *Clu* and *Anax1*. RevCSCs have a high tendency to differentiate to repopulate the colon in response to environmental stress [47,48]. Additionally, RevCSCs have been linked to poor response to chemotherapy [48,63]. Our data suggest that the expression of *BRAF^V600E^* pushes tumor stem cells toward RevCSCs to enhance colon tumor differentiation. Another group demonstrated that signaling from fibroblasts in the tumor microenvironment polarizes the stem cells toward RevCSCs [49]. Future studies should investigate the effect of activation of *BRAF* on fibroblast-epithelial colon tumor communications and the potential contribution of RevCSCs to poor outcomes in patients with *BRAF^V600E^* CRC. Overall, our data suggest that activation of *BRAF* shifts the stem-differentiation balance toward a differentiated cell state.

Loss of MSH2 is associated with microsatellite instability (MSI) in CRC [8], and we have previously shown that MSH2KO tumors were MSI as compared to Min tumors, which were microsatellite stable [12]. MSI CRC is characterized by poor differentiation [64,65]. Consistent with these findings, our data demonstrated that MSH2KO tumors have more tumor stem cells and less differentiated cells, such as goblet cells and enterocytes, compared to Min tumors. Additionally, MSH2KO cells had more directionality and speed toward the tumor stem cell population. We previously showed that ETBF-induced Min and MSH2KO tumors have the same level of β-catenin [12]. However, our scRNA-seq data revealed that MSH2KO tumor stem cells exhibit an increase in WNT signaling pathway and WNT-related genes such as *Axin2*, *Wnt6*, and *Wnt10a* compared to Min tumor stem cells. Additionally, organoids derived from MSH2KO tumors exhibited higher expression levels of *Axin2*, a WNT target gene [66], compared to Min organoids suggesting that the difference in gene expression is intrinsic to the tumor epithelial cells and not driven by other cells in the tumor microenvironment. The WNT signaling pathway is involved in maintaining the intestinal stem cell population and increased WNT signaling activity is associated with poorly differentiated CRC [67]. Therefore, we suggest that the increased WNT signaling activity in MSH2KO contributes to fewer differentiated cells in these tumors. It should be noted that, unlike our findings, data from the single-cell atlas of mismatch repair deficient (MMRd) and mismatch repair proficient (MMRp) human CRC [68] indicated that MMRp CRC had higher expression of WNT-target genes compared to MMRd CRC [68]. One possible explanation for this difference is that in our study we used murine colon tumors, which are pre-cancerous lesions, and loss of MMR may have a different effect on WNT pathway activity and stemness early in the tumorigenesis process than it does in cancer.

We also identified known and novel regulators that might be involved in the specification of different lineages. We found that BLM tumors showed a higher level of *Cdx2*-expressing cells compared to Min tumors, which was confirmed by using tumor-derived organoids. Additionally, BLM tumor tissue sections exhibited a higher level of CDX2 protein compared to Min tumor tissue. CDX2 is an intestinal transcription factor that is involved in intestinal development [53,54]. Loss of CDX2 in *BRAF* mutant CRC is associated with an increased stem cell population and increased oncogenic burden of the *BRAF* mutation [45,69,70]. Loss of CDX2 expression, usually through DNA methylation [71], concurrently with *BRAF* mutation is associated with poor prognosis in CRC patients [72,73]. Interestingly, we showed that knocking down Cdx2 significantly reduced the expression of differentiated lineages-related genes such as *Muc2*, *Atoh1*, and *Guca2a* in *BRAF* mutant tumor-derived organoids. Our data suggest that CDX2 induces the differentiation of BLM tumors. Additionally, consistent with low WNT activity in BLM tumors, CDX2 has been previously shown to suppress WNT signaling activity in CRC cells [74]. Interestingly, normal BLM and Min colon epithelium exhibited the same level of CDX2 protein whereas the level of CDX2 protein drastically decreased in Min colon tumors. It is possible that the expression of *BRAF*^V600E^ in Min mice maintains the expression of *Cdx2* in ETBF-induced colon tumors. Further investigation is required to study how *BRAF*^V600E^ regulates *Cdx2* expression in ETBF-induced colon tumors.

Paneth cells are known to be the source of the antimicrobial hormones, GUCA2A and REG3G, which play an essential role in intestinal homeostasis [75,76]. We previously demonstrated through bulk RNA-seq that ETBF induces an increase in *Reg3g* expression in BLM tumors compared to Min tumors [27]. Here we determined that as expected *Guca2a* and *Reg3g* were mainly expressed by Paneth cells in Min tumors. However, they were expressed in both secretory cells and mainly in enterocytes in BLM tumors. Our findings suggest that BLM tumors have altered colon epithelial function allowing for the production and possible secretion of antimicrobial hormones in multiple differentiated lineages in response to ETBF colonization. Additionally, we found that BLM tumors had a significant increase in the secretory-like population, which was characterized by the expression of *Saa3.* SAA3 was previously reported to ameliorate dextran sodium sulfate (DSS)-induced colitis and maintain the expression of antimicrobial peptides *Reg3g* and *Reg3b* [77] suggesting that secretory-like cells might be linked to the increased expression of the anti-microbial peptides in BLM tumors. The secretory-like population had also high enrichment for the EMT hallmark gene set. Therefore, it is possible that the increased differentiation in BLM tumors is accompanied by better colon homeostasis and enhanced tumor invasiveness through increasing the expression of anti-microbial peptides and EMT-related genes, respectively.

Our RNA velocity analysis suggested *Ndrg1* as a potential additional driver for BLM tumor differentiation. NDRG1 is associated with differentiation in other cancers and cell types [78]. Additionally, NDRG1 inhibits WNT activity by preventing the nuclear localization of β-catenin [79]. *Ndrg1* showed high-velocity expression in the tumor stem cell population of BLM tumors, while it was absent in Min tumor stem cells. This result suggests that low WNT activity in BLM tumors may be due to the high-velocity level of *Ndrg1* in these tumors. Other regulators for colon tumor differentiation were identified such as *Styl2*, *Kcnb2*, *Nrg1*, and *Mpp5*. Future work will be required to explore how Ndrg1 mechanistically regulates differentiation in BLM colon tumors.

## 5. Conclusions

Overall, we propose a model where high expression of ProCSCs markers and high activity of WNT signaling in MSH2KO tumors maintain the tumor stem cell population, resulting in more cycling stem cells in MSH2KO tumors and reducing their tendency to differentiate toward different lineages. However, increased RevCSCs marker expression with low WNT activity in BLM tumors increases the differentiation potential of BLM colon tumors. Furthermore, the increased expression of *Cdx2*, and other differentiation-driving transcription factors like *Klf4* and the WNT antagonist, *Ndrg1*, in BLM tumors push the tumor stem cells toward the various differentiated lineages (Supplemental Figure S6E). Determining how the differentiation of inflammation-associated colon tumor epithelial cells is regulated in different genetic backgrounds will lead to a greater understanding of tumor epithelial cell biology that has the potential to alter therapy response.

## 6. Limitations of the Study

In this study, we mainly focused on investigating the effect of genetic mutations on inflammation-induced murine colon tumor heterogeneity and differentiation. Future studies should apply our findings to human colon tumors and CRC samples. Additionally, we performed scRNA-seq on pooled tumors from a single mouse, which limits our ability to understand biological variability. While the scRNA-seq data generated for this study are robust, with more than 6000 cells passing quality control per sample, future work will require validating findings in additional biological replicates. We demonstrated that BLM tumors are more differentiated compared to Min and MSH2KO epithelial tumors. It is worth mentioning that MSH2KO and Min tumors were harvested from the distal part of the colon, while BLM tumors were predominantly harvested from the mid-proximal part of the colon. Although we previously showed that the location of BLM tumors within the colon (distal versus mid-proximal) had little effect on gene transcription by bulk-RNA-seq [27], we cannot rule out the effect of colon location on colon tumor differentiation in this study. Therefore, future studies should investigate the effect of colon location on inflammation-induced colon tumor cell heterogeneity/differentiation. Additionally, our scRNAseq data also contain information on immune cell and fibroblast populations present in colon tumors. Future research should investigate the effect of *BRAF* mutation and/or *Msh2* deletion on immune cell infiltration and activation and fibroblast-colon tumor epithelium interaction.

## Data Availability

All raw and processed sequencing data generated in this study were submitted to the NCBI Gene Expression Omnibus (GEO) under accession number GSE249625.

## Supplementary Materials

The following supporting information can be downloaded at: https://www.mdpi.com/article/10.3390/cancers16112040/s1, Figure S1. Violin plot of normalized gene expression of epithelial, fibroblast, lymphoid, and myeloid cell gene markers in the combined BLM, BLM2, Min, and MSH2KO scRNA-seq data. Figure S2. Heatmap of normalized gene expression of gene markers for stem cells, enterocyte cells (EC), transit-amplifying cells (TA), EC/brush border, goblet cells (GC), and Paneth cells (PC), and secretory-like cells in combined BLM, BLM2, Min, and MSH2KO colon epithelial tumors. Figure S3. Feature plot of normalized gene expression of different genes in BLM, BLM2, Min, and MSH2KO colon epithelial tumors. Figure S4. The diffusion map captures the differentiation trajectory of BLM tumors. Figure S5. Stem cells of BLM and MSH2KO colon epithelial tumors have low and high WNT signaling activity, respectively. Figure S6. *Cdx2* and *Ndrg1* are involved in BLM colon tumor epithelial cell differentiation. Table S1. Supplemental_Table_S1.xls. Table S2. Supplemental_Table_S2.xls. Table S3. TaqMan qPCR assays.
