# Supplementary material for: Single-Cell Profiling Reveals the Impact of Genetic Alterations on the Differentiation of Inflammation-Induced Murine Colon Tumors
Source: Cancers (Basel). 2024 May 28;16(11):2040. doi: 10.3390/cancers16112040 (PMC11171101; doi:10.3390/cancers16112040)
Supplement: Supplementary file 1 [file cancers-16-02040-s001.zip › Ghobashi et al Supplemental Material-MDPI.pdf]

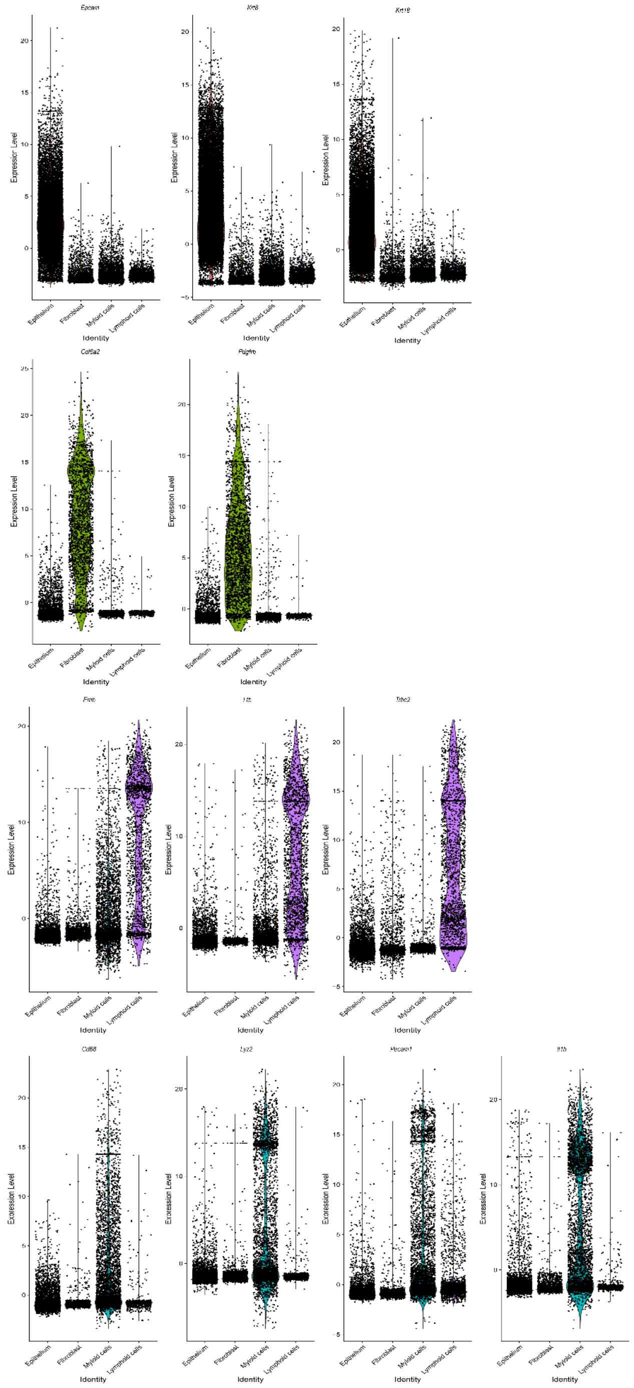

Supplemental Figure S1. Violinplot of normalized gene expression of epithelial, fibroblast, lymphoid, and myeloid cell gene markers in the combined BLM, BLM2, Min, and MSH2KO scRNA-seq data.

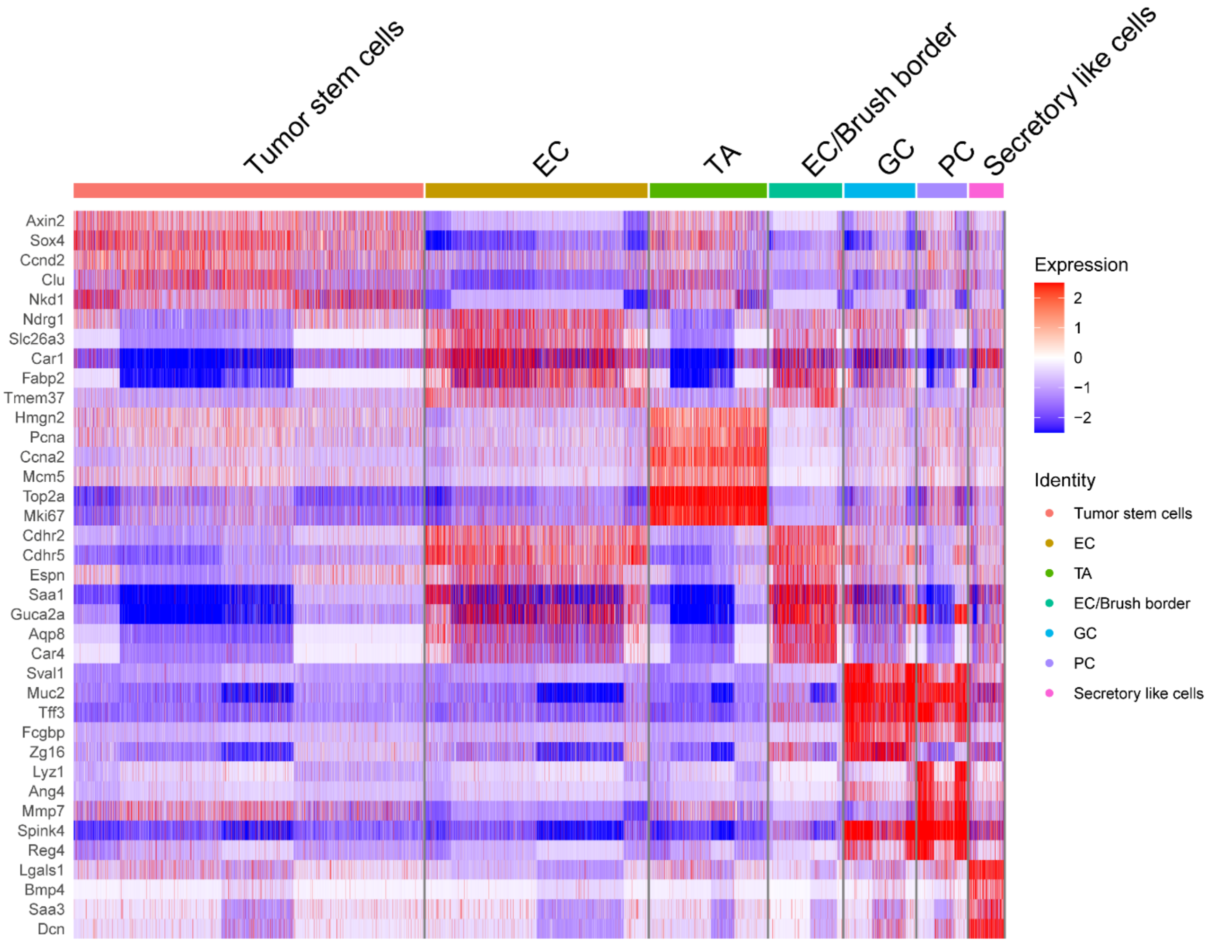

Supplemental Figure S2. Heatmap of normalized gene expression of gene markers for tumor stem cells, enterocyte cells (EC), transit-amplifying cells (TA), EC/brush border, goblet cells (GC), and Paneth cells (PC), and secretory-like cells in combined BLM, BLM2, Min, and MSH2KO colon epithelial tumors.

A

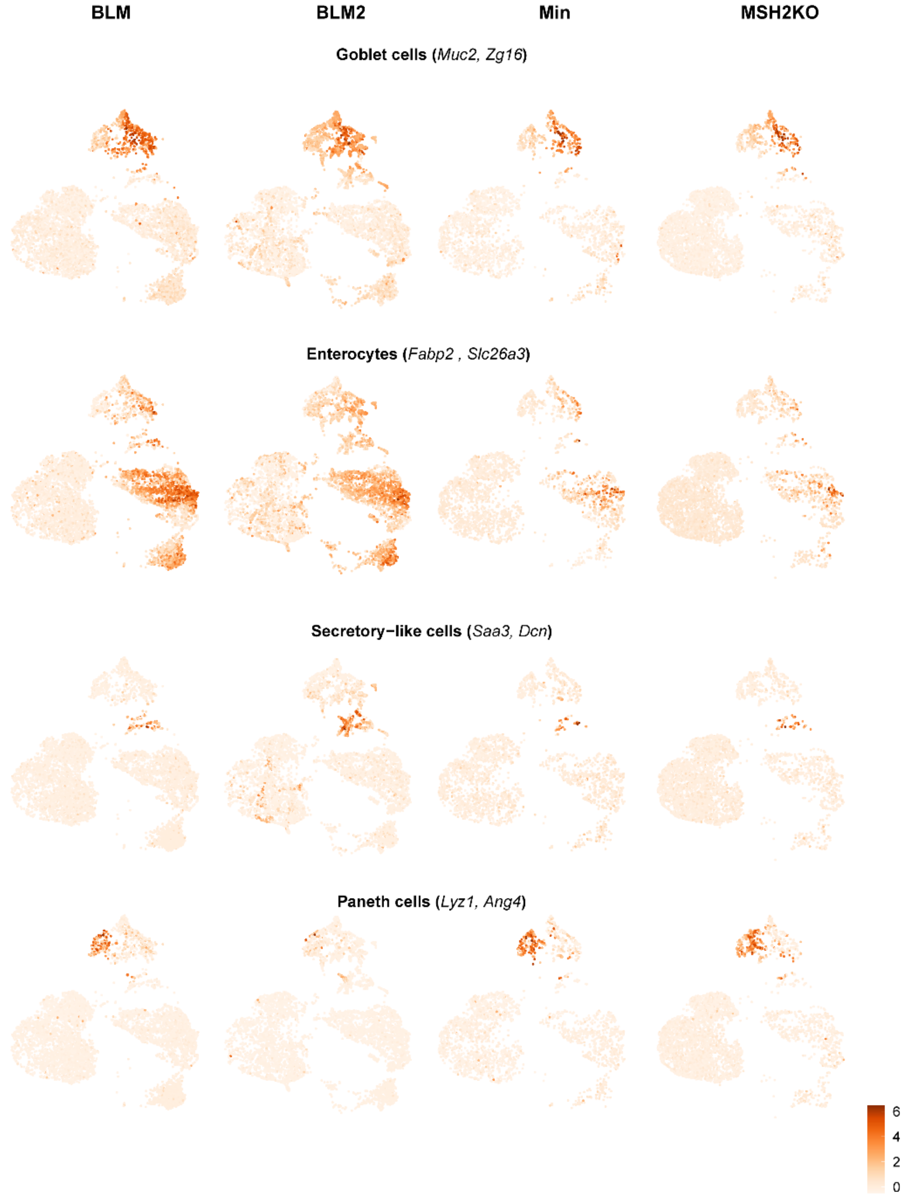
BLM
BLM2
Min
MSH2KO
Goblet cells (Muc2, Zg16)
Enterocytes (Fabp2 , Slc26a3)
Secretory−like cells (Saa3, Dcn)
Paneth cells (Lyz1, Ang4)
6
4
2
0

B

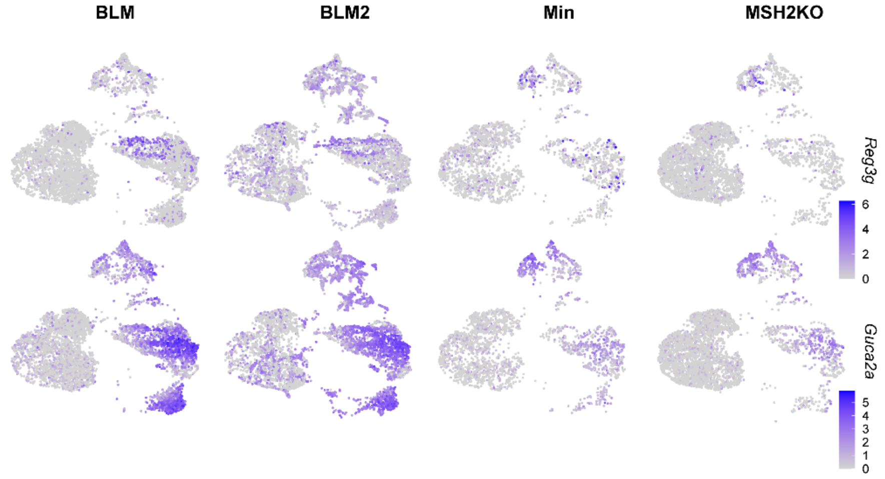
BLM
BLM2
Min
MSH2KO
Reg3g
6
4
2
0
Guca2a
5
4
3
2
1
0

Supplemental Figure S3. Gene expression in colon epithelial tumors. (A) Uniform manifold approximation and projection (UMAP) plot of BLM, BLM2, Min, and MSH2KO tumor epithelial scRNA-seq data showing the expression of different cluster markers. Color gradient represents the average gene expression. (B) UMAP plot of BLM, BLM2, Min, and MSH2KO tumor epithelial scRNA-seq data showing the expression of indicated genes. Color intensity represents normalized gene expression.

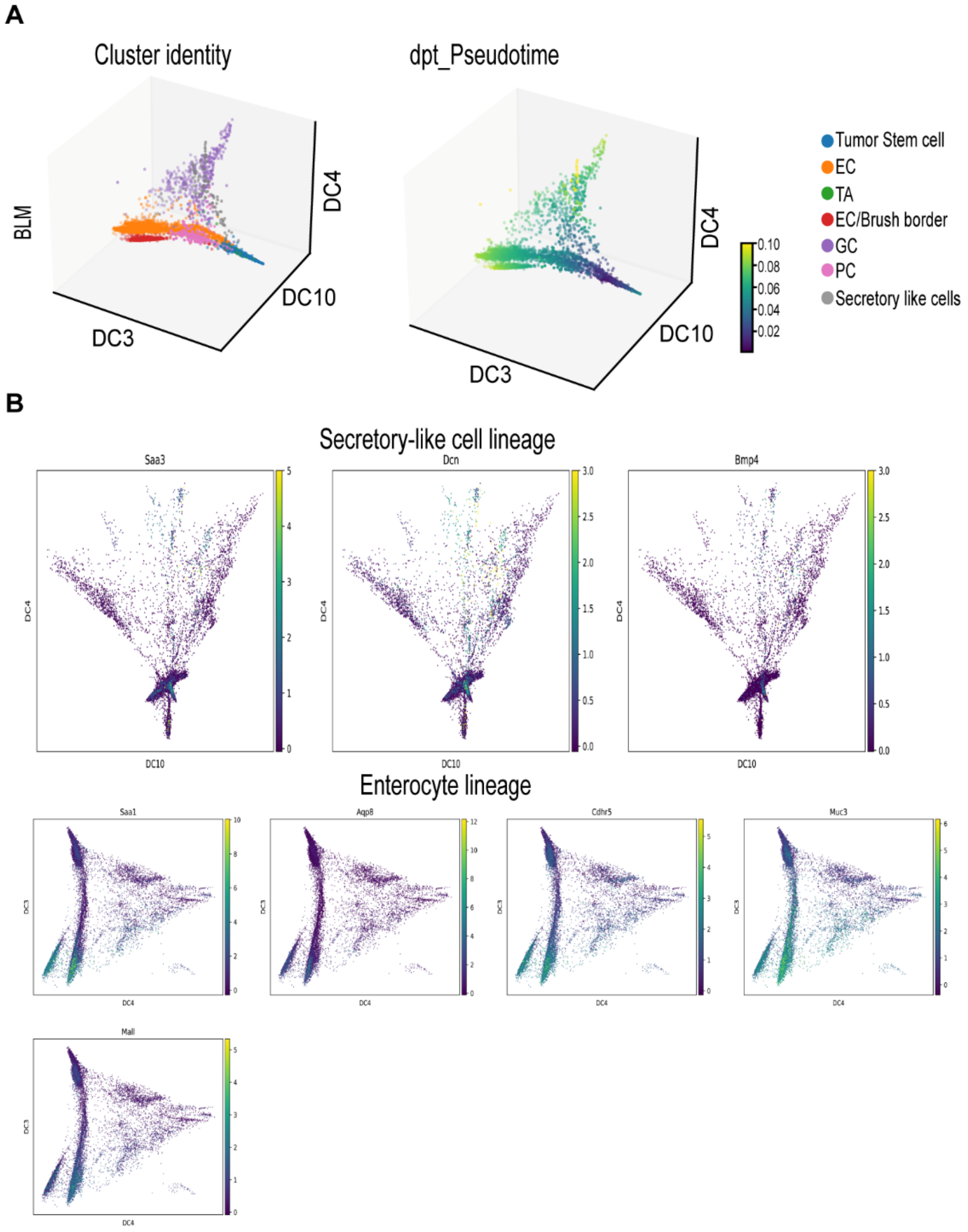

Supplemental Figure S4. The diffusion map captures the differentiation trajectory of BLM tumors. (A) The diffusion-map embedding of BLM colon epithelial tumor is colored by cell type (left, Cluster identity) and diffusion pseudotime (right, dpt_Pseudotime) (enterocyte cells (EC), transit-amplifying cells (TA), goblet cells (GC), Paneth cells (PC)). The colors represent represent pesudotime value. (B) Expression of regional markers of indicted cell lineages in combined BLM2, BLM, Min, and MSH2KO colon epithelial tumors. Colors represent normalized gene expression.

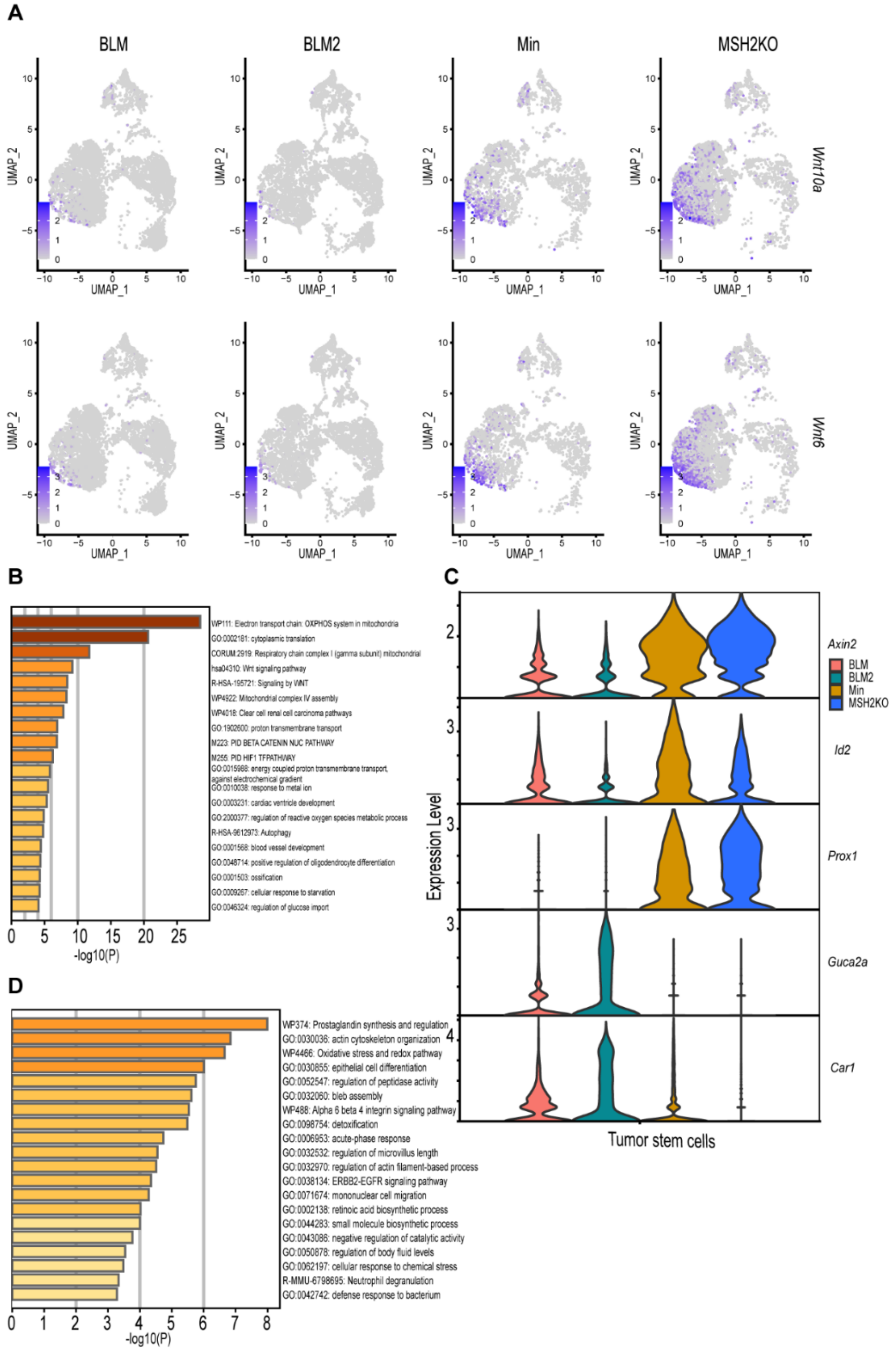

Supplemental Figure S5. Tumor stem cells of BLM and MSH2KO colon epithelial tumors have low and high WNT signaling activity, respectively. (A) Featureplot of normalized gene expression of *Wnt10a* and *Wnt6* in BLM, BLM2, Min, and MSH2KO colon epithelial tumors. Color intensity represents normalized gene expression. (B) Functional gene annotations for differentially expressed genes (DEGs) in the tumro

stem cell populations of MSH2KO versus Min generated by Metascape. (C) Violinplot of normalized gene expression of indicated genes in stem cell clusters of BLM, BLM2, Min, MSH2KO colon epithelial tumors. (D) Functional gene annotations for DEGs in the stem cell populations of BLM2 versus Min generated by Metascape.

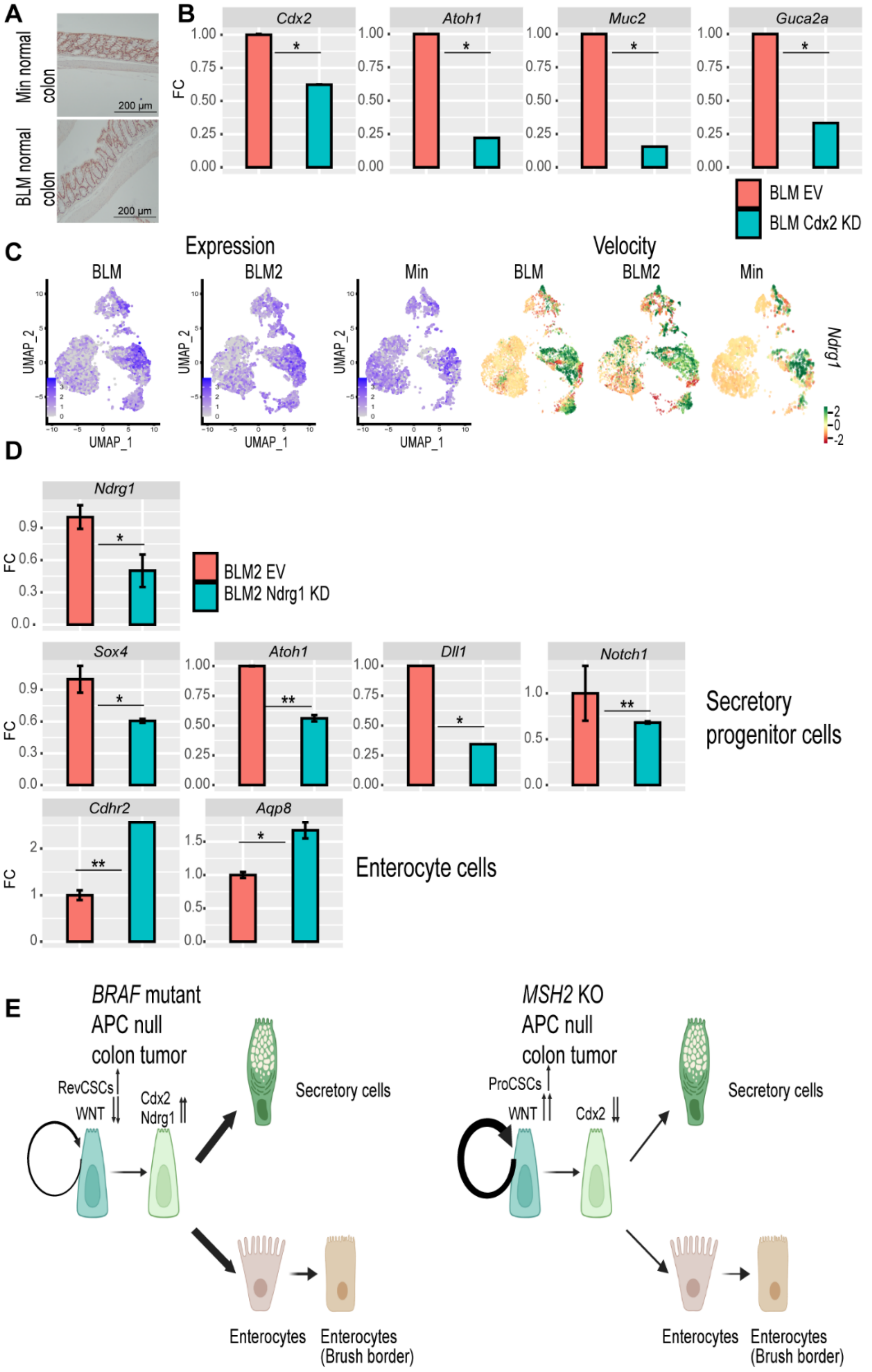

Supplemental Figure S6. *Cdx2* and *Ndrg1* are involved in BLM colon tumor epithelial cell differentiation.

(A) Representative CDX2 IHC in BLM normal colon and Min normal colon (Scale bar, 200 µm). (B) Gene

expression of the indicated genes by RT-qPCR in BLM organoids with empty vector (EV) or Cdx2 knockdown (KD). Expression of all the genes was normalized to the housekeeping gene *Ppia* and then to BLM EV organoids. Results are represented as the mean of 3 biological replicates +/− SEM. (C) Featureplot of *Ndrg1* expression (left) and velocity (right) in BLM, BLM2, and Min colon epithelial tumors. Colors represent normalized gene and velocity expression. (D) Gene expression of the indicated genes by RT-qPCR in BLM2 organoids with EV or Ndrg1 knockdown (KD). Expression of all the genes was normalized to the housekeeping gene *Ppia* and then to EV. Results are represented as the mean of 3 biological replicates +/− SEM. (E) The model in which high expression of differentiation-driving transcription factors such as *Cdx2* and *Ndrg1* promotes the differentiation of BLM colon tumors while increased WNT signaling activity increases tumor stem cells in MSH2KO colon tumors. Significance was determined by paired t-test. *p <= 0.05, **p <= 0.01.

Gene	Taqman assay
cdx2	Mm01212280_m1
Ndrg1	Mm07295892_m1
Atoh1	Mm00476035_s1
Muc2	Mm01276696_m1
Dcn	Mm00514535_m1
Aqp8	Mm00431846_m1
Notch1	MM00627185_M1
Sox4	Mm00486320_s1
Dll1	Mm01279269_m1
Lgr5	Mm00438890_m1
Axin2	Mm00443610_m1
Guca2a	Mm00433863_m1
Cdhr2	Mm01344960_m1
Ppia	Mm02342430_g1

Supplemental Table S3. Taqman qPCR assays
